# PEX5, a novel target of microRNA-31-5p, increases radioresistance in hepatocellular carcinoma by activating Wnt/β-catenin signaling and homologous recombination

**DOI:** 10.7150/thno.42371

**Published:** 2020-04-06

**Authors:** Jie Wen, Kai Xiong, Abudureyimujiang Aili, Hao Wang, Yuequan Zhu, Zhengquan Yu, Xueyan Yao, Ping Jiang, Lixiang Xue, Junjie Wang

**Affiliations:** 1 Peking University Third Hospital, Beijing 100191, China.; 2 Beijing Luhe Hospital, Capital Medical University, Beijing 101149, China.; 3 College of Biological Sciences, China Agricultural University, Beijing 100193, China.; 4 Peking University People's Hospital, Beijing 100044, China.

**Keywords:** miR-31-5p, PEX5, β-catenin, Homologous Recombination, Radiosensitivity

## Abstract

**Rationale**: Hepatocellular carcinoma (HCC) is the second leading cause of cancer-related death worldwide, with high recurrence and metastasis rates. Although radiation is an effective treatment for tumors, it is often limited by intrinsic radioresistance in HCC. The contributions of dysregulated microRNAs, including miR-31-5p, to HCC progression have been recently reported. However, the role of miR-31-5p in the radiation response of HCC is unknown. In this study, we aimed to investigate the impact of miR-31-5p on HCC radiosensitivity.

**Methods**: miR-31-5p expression in HCC tissues, paired adjacent tissues, and HCC cell lines was measured using quantitative real-time polymerase chain reaction and *in situ* hybridization. Bioinformatic analyses, gain- and loss-of-function experiments, and luciferase reporter assays were performed to validate peroxisomal biogenesis factor 5 (PEX5) as a direct target of miR-31-5p. The biofunctions of PEX5 and miR-31-5p in HCC were determined by Transwell, wound-healing, and Cell Counting Kit-8 (CCK8) assays. A colony formation assay was used to evaluate the radiosensitivity of HCC cells. The interaction among PEX5, β-catenin, Rac1, and JNK-2 was confirmed by coimmunoprecipitation. A xenograft tumor model was established to validate the effects of miR-31-5p and PEX5 on HCC progression and radiosensitivity *in vivo.*

**Results:** Low expression of miR-31-5p in HCC specimens, as observed in this study, predicted a poor clinical outcome. However, the expression pattern of PEX5, as a direct target of miR-31-5p, was opposite that of miR-31-5p, and high PEX5 expression indicated poor prognosis in HCC patients. Ectopic expression of PEX5 increased the proliferation, migration, and invasion abilities and enhanced the radioresistance of HCC cells *in vitro* and* in vivo*; however, these phenotypes were inhibited by miR-31-5p. Mechanistically, PEX5 stabilized cytoplasmic β-catenin and facilitated β-catenin nuclear translocation to activate Wnt/β-catenin signaling. Moreover, upon radiation exposure, PEX5 reduced excessive reactive oxygen species (ROS) accumulation and activated the homologous recombination (HR) pathway, which protected HCC cells from radiation-induced damage.

**Conclusions**: Our findings demonstrated a novel role for PEX5 as a miR-31-5p target and a mediator of the Wnt/β-catenin signaling and HR pathways, providing new insights into studying HCC radiation responses and implicating PEX5 and miR-31-5p as potential therapeutic targets in HCC.

## Introduction

Liver cancer is the second most common cause of cancer-related mortality, and hepatocellular carcinoma (HCC) accounts for over 90% of liver cancer cases 1. Most patients with HCC are diagnosed at late stages, resulting in high mortality 2. Radiotherapy (RT) is considered a common treatment for HCC because patients usually exhibit high resistance to chemotherapy and low success rates of radical surgery 3. Many reports have shown that RT significantly improves the clinical prognoses of patients, including those with metastatic HCC 4 and unresectable HCC 5. However, owing to endogenous radioresistance in some patients with HCC 6, the efficacy of RT has historically been limited. Hence, identifying potential targets that could reduce radioresistance while protecting " innocent bystander " tissues against radiation-induced damage without compromising tumor radiosensitivity in HCC patients is of great interest.

Recent reports have indicated that miR-31-5p is aberrantly expressed in many cancers, including HCC, and regulates cancer progression and chemoradiotherapeutic responses 7, 8. miR-31-5p promotes the chemosensitivity of breast cancer cells by suppressing the NF-κB 9 and AKT signaling pathways 10. Importantly, miR-31-5p also plays an important role in regulating radioresistance in esophageal adenocarcinoma 11 and colon cancer, as shown in our previous work 12. Radiation of whole blood samples from healthy donors significantly increased the level of miR-31-5p in released extracellular vesicles 13, further suggesting the importance of miR-31-5p in regulating radioresistance. However, the function and molecular mechanisms of miR-31-5p in regulating the radiation response of HCC are still unclear.

Radioresistant cancer cells can protect themselves by enhancing the DNA repair response 14. The response to radiation-induced DNA double-strand breaks involves two common DNA damage response pathways: homologous recombination (HR) and nonhomologous end joining (NHEJ). HR is an error-free repair pathway and is active only during the S and G2 phases, while NHEJ performs more rapidly and is active throughout the cell cycle 15. If DNA breaks are neither repaired nor removed, the DNA damage response triggers cell death. Activation of the canonical Wnt/β-catenin pathway can promote the DNA damage repair process 16, consequently desensitizing cancer cells to radiation and chemotherapy 17-19. Interestingly, many reports have indicated that miR-31-5p regulates the Wnt/β-catenin signaling pathway in a context-dependent manner. miR-31-5p can directly target DKK1 and AXIN1 to activate Wnt/β-catenin signaling in mammary stem cells 20, lung cancer cells 21 and osteosarcoma cells 22. On the other hand, miR-31 suppresses Wnt/β-catenin signaling in mesenchymal stem cells by targeting Frizzled-3 23. However, the regulatory role of miR-31-5p in mediating Wnt signaling and tumor radioresistance in HCC remains unknown.

Peroxisomal biogenesis factor 5 (PEX5), plays an essential role in peroxisomal function. PEX5 functions as a receptor for transport peroxisomal matrix proteins in peroxisomes upon intercellular stress to modulate redox homeostasis 24. As an essential component of peroxisome function, PEX5 is predominantly involved in peroxisomal protein import 24. Peroxisomes contribute to the metabolism of reactive oxygen species (ROS) and have been linked to aberrant metabolic processes in cancer 25. In delivering abundant ROS-scavenging enzymes such as catalases into the peroxisome, PEX5 protects cells from ROS-induced stress. Cai et al. reported that PEX5 knockdown inhibited the import of catalase into peroxisomes, augmented cellular ROS accumulation, and consequently suppressed HCC cell growth 26. Defects in PEX5 could lead to peroxisome starvation, ROS accumulation, and apoptosis 27. However, whether PEX5 regulates radioresistance in HCC is entirely unknown.

Here, we utilized both *in vitro* and *in vivo* models to demonstrate that miR-31-5p enhanced the radiosensitivity of cancer cells in HCC by directly suppressing PEX5, which concomitantly modulated the Wnt/β-catenin and HR signaling pathways.

## Materials and methods

### Statistical analysis

Each experiment was performed at least three times, and data are expressed as the mean ± S.D. values. Data analysis was performed using GraphPad Prism. The statistical significance of differences among groups was assessed by Student's *t*-test or one-way analysis of variance. The χ^2^ test was applied to analyze the associations among miR-31-5p expression, PEX5 expression, and clinical features. The χ2 test was also used to analyze the incidence of lung metastasis in xenografted nude mice and compare the miR-31-5p expression level between HCC patients with or without metastasis. A rank sum test was applied to analyze data that did not conform to a normal distribution. The Kaplan-Meier method was used for survival analysis. A value of p < 0.05 was considered significant.

Details of the methods and materials used are provided in the Supplementary Materials and Methods.

## Results

### miR-31-5p is downregulated in HCC tissues and is negatively associated with HCC clinical outcomes

**Figure 1:**
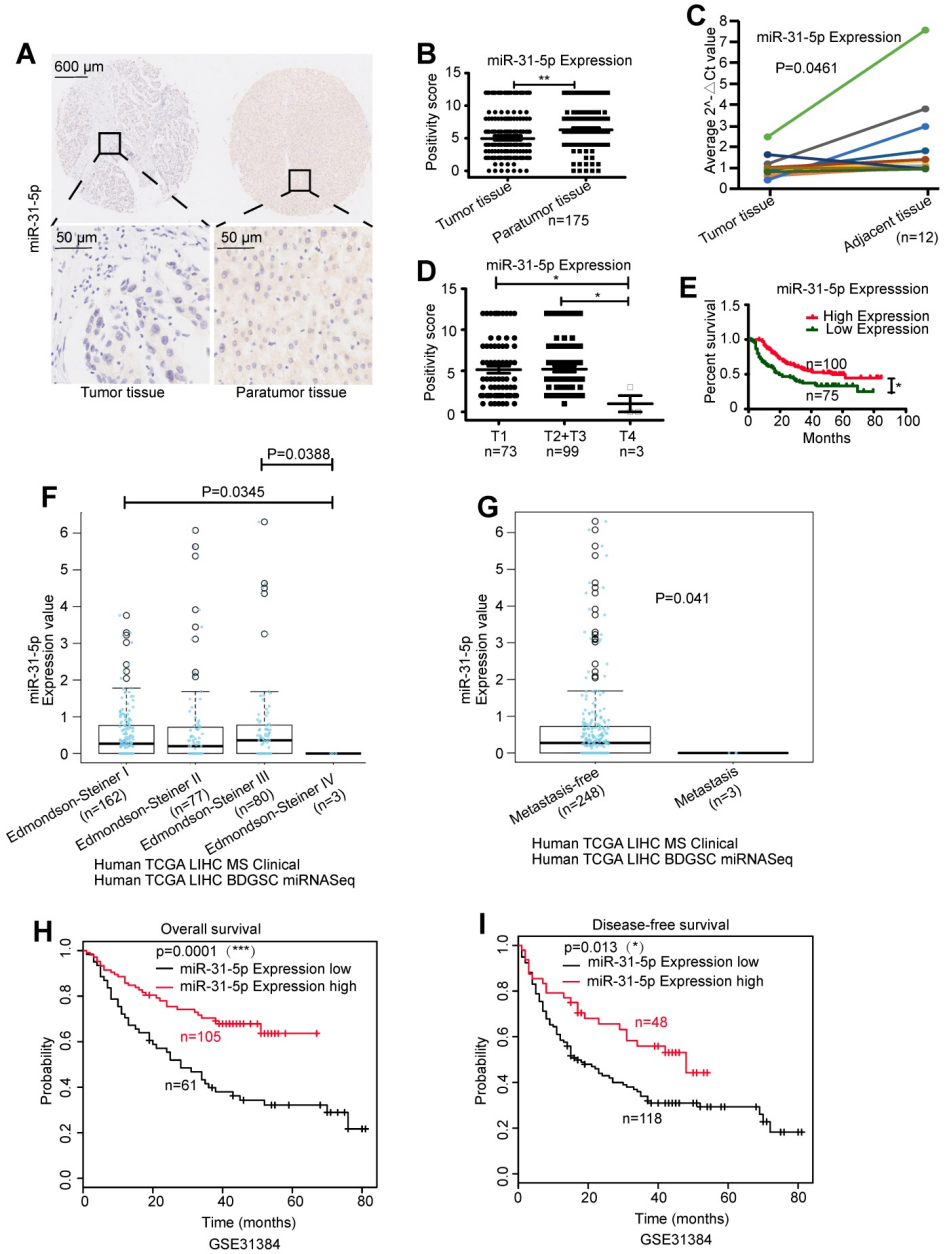
miR-31-5p was downregulated in HCC tissues. (A-B) miR-31-5p was downregulated in HCC tissues, as demonstrated using ISH with a tissue microarray (n = 175, **P < 0.01). (C) miR-31-5p expression in paired HCC specimen. (D) Association between the miR-31-5p level and HCC clinical stage. miR-31-5p was downregulated in patients with advanced HCC compared to patients with early HCC (n = 175, *P < 0.05). (E) HCC patients with low expression of miR-31-5p had poor clinical outcomes (n = 175, *P < 0.05). (F) miR-31-5p expression in patients with tumors of different clinicopathological grades (Edmondson-Steiner grade, LinkedOmics database). As shown, the patients with high-grade tumors had low miR-31-5p expression levels. (G) miR-31-5p expression in HCC patients with or without metastasis. (LinkedOmics database, TCGA data). The patients with metastasis had lower expression of miR-31-5p than the patients without metastasis. (H-I) Kaplan-Meier survival curve for patients in the GSE31384 dataset. Overall survival and disease-free survival were markedly decreased in HCC patients with low miR-31-5p expression. (P=0.001, P=0.013; Kaplan-Meier survival analysis). Abbreviations: LIHC, liver hepatocellular carcinoma; T, tumor tissues; N, normal tissues.

To investigate the role of miR-31-5p in HCC, we measured its expression level in an HCC tissue microarray and correlated these values with patients' prognoses. The expression level of miR-31-5p was decreased by 17.6% in HCC tumor tissues compared to the corresponding paratumor tissues (Figure 1A-B). Downregulation of miR-31-5p in HCC was further confirmed in 12 paired HCC specimens (Figure 1C). Further, miR-31-5p expression was negatively correlated with the T stage, i.e., tumors in advanced stages had low miR-31-5p levels (Figure 1D). The miR-31-5p expression level was significantly decreased in patients whose tumors had diameters exceeding 5 cm or exhibited vascular invasion (hepatic vein, portal vein or other blood vessels) (Table S3), suggesting a negative correlation between miR-31-5p expression and both tumor size and vascular invasion. Consistent with this finding, patients with lower miR-31-5p levels had shorter overall survival times (Figure 1E). Moreover, data from LinkedOmics showed that miR-31-5p levels were lower in HCC patients with high-grade tumors (Edmondson-Steiner grade) or metastasis than in HCC patients with low-grade or nonmetastatic tumors (Figure 1F-G). Further, the poor overall survival (Figure 1H) and disease-free survival (Figure 1I) prognoses of HCC patients with low expression levels of miR-31-5p were validated using data from GSE31384. Collectively, these results showed that miR-31-5p levels are low in HCC patients and suggested that miR-31-5p expression is negatively associated with clinical prognosis, TNM stage, pathological grade, metastasis, vascular invasion, and tumor size.

### miR-31-5p decreases HCC radioresistance *in vitro*

**Figure 2:**
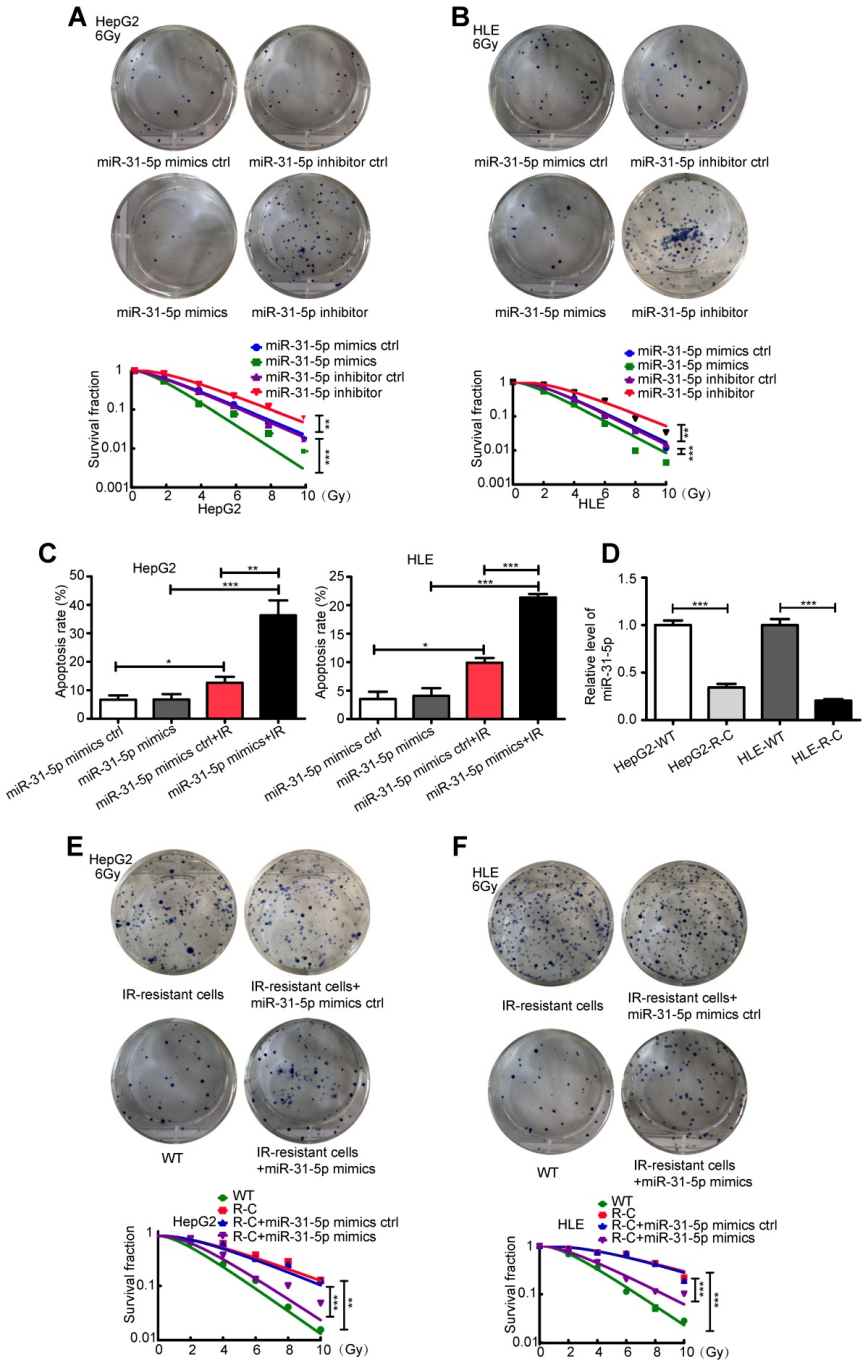
miR-31-5p sensitized HCC to radiation *in vitro*. (A-B) Radiation sensitivity was tested with colony formation assays in both cell lines with different levels of miR-31-5p. In both cell lines, miR-31-5p downregulation increased colony formation upon exposure to 6 Gy irradiation, and miR-31-5p upregulation decreased colony formation upon exposure to 6 Gy irradiation. Top: Representative images of colony formation assays. Bottom: Statistical analysis results (**P < 0.01, ***P < 0.001). (C) The apoptosis rate of HepG2 and HLE cells with different miR-31-5p levels upon irradiation was validated by flow cytometry (*P < 0.05, **P < 0.01, ***P < 0.001). (D) The levels of miR-31-5p in HepG2-R-C and HLE-R-C were validated by qRT-PCR (***P < 0.001). (E-F) Radiation sensitivity of HepG2-R-C and HLE-R-C with different levels of miR-31-5p. HepG2 R-C and HLE R-C formed more colonies upon exposure to 6 Gy radiation than HepG2 WT and HLE WT cells, and this pattern was reversed by the miR-31-5p mimics. Top: Representative images of colony formation assays. Bottom: Statistical analysis results (**P < 0.01, ***P < 0.001). All of the above results are representative of three independent experiments. Abbreviations: ctrl, control; WT, wild-type; R-C, radiotherapy-resistant cells; IR, irradiation; GAPDH, glyceraldehyde 3-phosphate dehydrogenase.

To further investigate the clinical correlation of miR-31-5p expression, we studied the effect of miR-31-5p on the response to radiation in two hepatoma cell lines, HepG2 and HLE. As shown in Figure 2A-B, miR-31-5p knockdown desensitized HepG2 and HLE cells to radiation; however, miR-31-5p upregulation produced the opposite effect. Consistent with these results, a miR-31-5p mimics notably increased apoptosis in both cell lines after irradiation (Figure S1A-B, Figure 2C). Further, remarkably low levels of miR-31-5p were observed in radiation-resistant HepG2 cells (HepG2-R-C) and in radiation-resistant HLE cells (HLE-R-C) compared to those in HepG2 and HLE wild-type (WT) cells (Figure 2D). The miR-31-5p mimics partially resensitized HepG2-R-C and HLE-R-C to radiation (Figure 2E-F). These results suggest that miR-31-5p modulates the radiosensitivity of HCC cells.

### PEX5, a novel target of miR-31-5p, is upregulated in HCC, with a positive clinical correlation

**Figure 3:**
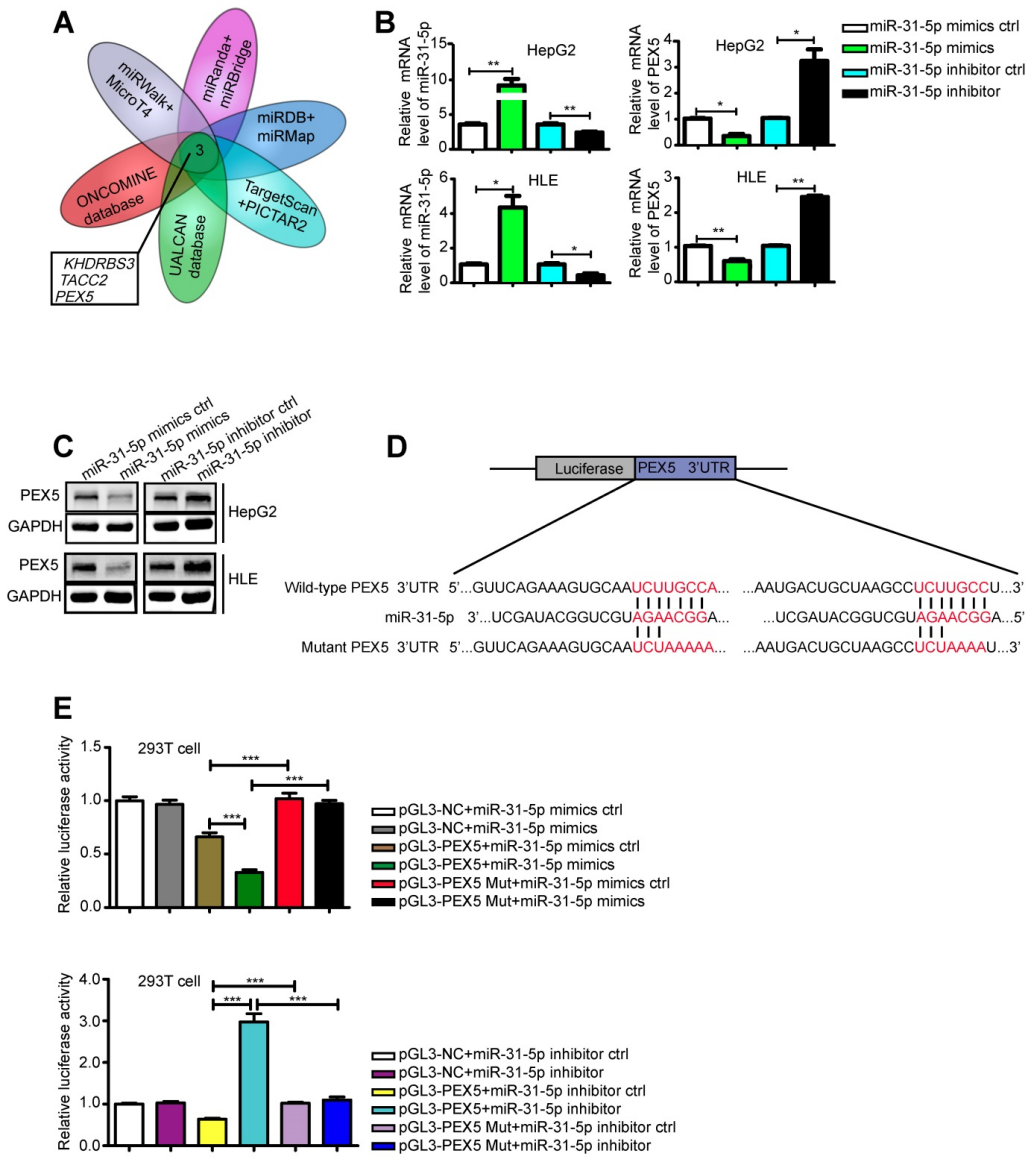
PEX5 was a direct target of miR-31-5p in HCC. (A) Venn diagram of the results of integrated analysis of miRNA datasets and cancer databases. (B-C) PEX5 was downregulated by the miR-31-5p mimics, as validated by qRT-PCR and WB (*P < 0.05, **P < 0.01). The results are representative of three independent experiments. (D) Schematic diagrams of miR-31-5p, its putative binding sequence in the 3'-UTR of PEX5 and the mutant PEX5 3'-UTR. (E) The luciferase activity of the PEX5 3'-UTR was reduced by the miR-31-5p mimics and increased by the miR-31-5p inhibitor; however, mutation of the 3'-UTR of PEX5 reversed these trends (***P < 0.001). Abbreviations: ctrl, control; GAPDH, glyceraldehyde 3-phosphate dehydrogenase; mut, mutant.

miRNAs function by regulating their target genes. Therefore, we screened various databases for possible targets of miR-31-5p and identified *KHDRBS3*, *TACC2*, and *PEX5* as potential targets (Figure 3A). Indeed, we confirmed that the miR-31-5p mimics downregulated the mRNA expression levels of *KHDRBS3*, *TACCA* and *PEX5*, whereas a miR-31-5p inhibitor promoted the expression of these genes (Figure S2A-C, Figure 3B). However, based on the Integrative Molecular Database of Hepatocellular Carcinoma (HCCDB), we further found that the mRNA level of only *PEX5* affected the overall survival of HCC patients (Figure S2D-F). In addition, *PEX5* was identified as the target of miR-195, which was found to be downregulated in HCC 28. We thus selected *PEX5* as our target gene of interest. The miR-31-5p mimics inhibited PEX5 protein expression, while the miR-31-5p inhibitor reversed this effect (Figure 3C). A luciferase assay showed that PEX5 3′-UTR activity was repressed by the miR-31-5p mimics and increased by the miR-31-5p inhibitor; however, no significant influence on these reporters was observed when the putative miR-31-5p binding site on PEX5 was mutated (Figure 3D-E), indicating that PEX5 is a direct target of miR-31-5p in HCC.

**Figure 4:**
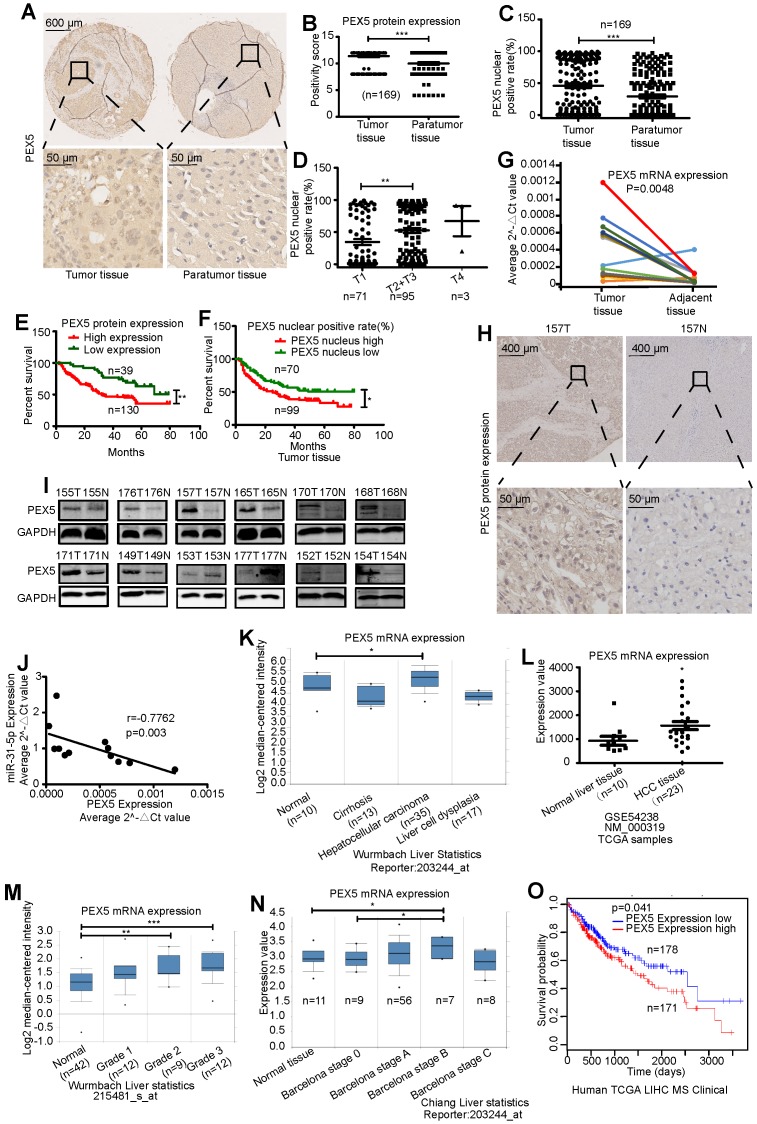
PEX5 was upregulated in HCC tissues. (A-B) The PEX5 protein level was upregulated in HCC tissue, as shown by immunohistochemical analysis of a tissue microarray (n = 169, ***P < 0.001). (C) Nuclear positive rate of PEX5 in HCC tissues and paratumor tissues (n = 169, ***P < 0.001). (D) Association between the nuclear positive rate of PEX5 and clinical stage of HCC (n = 169, **P < 0.01). (E-F) Association between the protein level or nuclear positive rate of PEX5 and overall survival (n = 169, *P < 0.05, **P < 0.01). (G) The PEX5 mRNA level in 12 paired HCC specimens was measured by qRT-PCR (n = 12). (H) Representative image of PEX5 IHC in a sample from patient 157. The nuclear and cytoplasmic levels of PEX5 in HCC tissue were higher than those in normal tissue. (I) PEX5 protein level in 12 paired HCC specimens, as shown by WB. (J) Negative relationship between the PEX5 and miR-31-5p expression levels (Spearman correlation analysis, r=-0.7762, P=0.003). (K) High mRNA expression of PEX5 in HCC tissues (ONCOMINE database) (*P<0.05). (L) High mRNA expression of PEX5 in HCC tissues (GEO dataset) (*P<0.05). (M) PEX5 mRNA expression levels in HCC patients with tumors of different pathological grades (Edmondson-Steiner Grade, ONCOMINE database) (**P<0.01, ***P<0.001). (N) PEX5 mRNA expression levels in HCC patients at different clinical stages. (Barcelona Clinic Liver Cancer stage, ONCOMINE database) (*P<0.05). Patients with advanced-stage disease had high PEX5 mRNA levels. (O) Overall survival of HCC patients with high and low PEX5 expression levels. (P=0.041; LinkedOmics database, Kaplan-Meier survival analysis). Patients with high PEX5 levels had poor survival outcomes. Abbreviations: T, tumor tissues; N, normal tissues; GAPDH, glyceraldehyde 3-phosphate dehydrogenase.

To assess the clinical correlation of PEX5 expression in HCC, we first measured the PEX5 protein expression level using a tissue microarray (Figure 4A-B). PEX5 protein expression was significantly increased in tumor tissues compared to paratumor tissues. Interestingly, the nuclear positive rate of PEX5 in HCC tissues was significantly higher than that in adjacent tissues (Figure 4C). Patients with advanced-stage disease had relatively high nuclear positive rates of PEX5 (Figure 4D). Furthermore, PEX5 protein expression was significantly increased in patients with vascular invasion and a histological grade of ≥ 2 (Table S3). Importantly, patients with higher PEX5 protein expression levels showed lower survival rates (Figure 4E). In addition, higher nuclear positive rates of PEX5 correlated with poorer clinical outcomes (Figure 4F).

High expression of PEX5 in HCC tissues was confirmed in 12 paired HCC specimens (Figure 4G-I), and *PEX5* mRNA expression was inversely correlated with miR-31-5p expression (Figure 4J). Moreover, high mRNA levels of *PEX5* in HCC were found in ONCOMINE and Gene Expression Omnibus (GEO) datasets (Figure 4K-M). In the ONCOMINE datasets, *PEX5* mRNA was upregulated at the advanced stage (Barcelona Clinic Liver Cancer stage) of HCC (Figure 4N). We further verified the correlation of high PEX5 expression with poor survival of HCC patients using data from the LinkedOmics database (Figure 4O). Collectively, our findings indicate that PEX5 is upregulated in HCC patients and that its expression is positively associated with clinical outcome, pathological grade, clinical stage, and vascular invasion.

### PEX5 increases the radioresistance of HCC cells *in vitro* and* in vivo*

The effect of PEX5 on the response to radiation has not been reported. As PEX5 is a target of miR-31-5p, we speculated that it might also modulate the sensitivity of HCC cells to irradiation. To examine whether PEX5 mediates the effect of miR-31-5p on the radiation response, we cotransfected the miR-31-5p mimics with a PEX5 overexpression plasmid and observed that miR-31-5p-mediated reduction in the PEX5 level in HCC cells was rescued by the PEX5 overexpression plasmid (Figure S3A-B).

**Figure 5:**
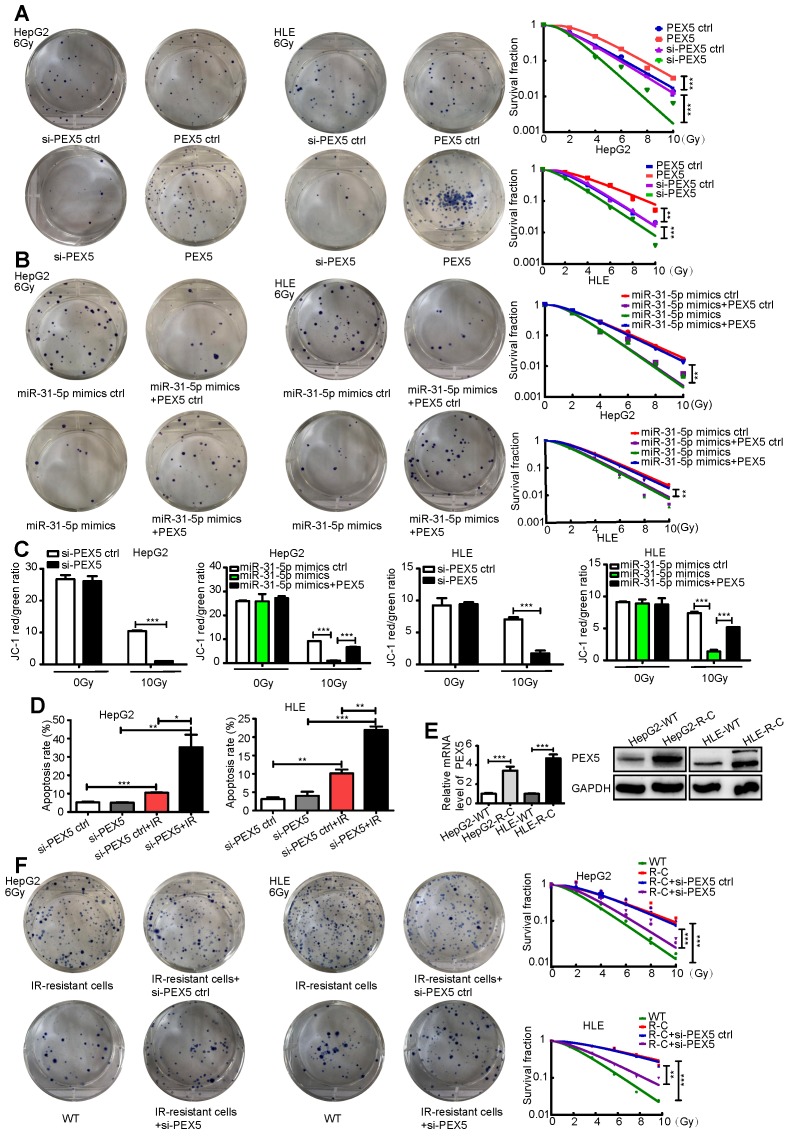
PEX5 desensitized HCC to radiation *in vitro*. (A) Radiation sensitivity was tested with colony formation assays in both cell lines with different levels of PEX5. Left: Representative images of colony formation assays. Right: Statistical analysis results (**P < 0.01, ***P < 0.001). (B) The miR-31-5p-mediated radiosensitization of HCC cells was abolished by PEX5. Left: Representative images of colony formation assays. Right: Statistical analysis results (**P < 0.01). (C) JC-1 levels in HepG2 and HLE cells with different levels of miR-31-5p or PEX5 expression with or without radiation exposure. (***P < 0.001). (D) The apoptosis rate of irradiated HepG2 and HLE cells with different PEX5 levels was validated by flow cytometry (*P < 0.05, **P < 0.01, ***P < 0.001). (E) The levels of PEX5 in HepG2-R-C and HLE-R-C were validated by WB and qRT-PCR (***P < 0.001). (F) Radiation sensitivity of HepG2-R-C and HLE-R-C with different levels of PEX5 expression. HepG2 R-C and HLE R-C formed more colonies upon exposure to 6 Gy radiation than HepG2 WT and HLE WT cells, and this trend was reversed by si-PEX5. Left: Representative images of colony formation assays. Right: Statistical analysis results (**P < 0.01, ***P < 0.001). All of the above results are representative of three independent experiments. Abbreviations: ctrl, control; si-PEX5, siRNA-PEX5; GAPDH, glyceraldehyde 3-phosphate dehydrogenase; WT, wild-type; R-C, radiotherapy-resistant cells; IR, irradiation.

As shown in Figure 5A-B, PEX5 upregulation desensitized HepG2 and HLE cells to radiation, an effect that was reversed by si-PEX5. Furthermore, the miR-31-5p-mediated increase in radiosensitivity was reversed by PEX5 overexpression. Upon irradiation, silencing PEX5 expression resulted in marked apoptosis in both cell lines, and the increase in apoptosis induced by miR-31-5p was abolished by PEX5 (Figure S3C-D, Figure 5C-D). Further, obviously high levels of PEX5 were observed in HepG2-R-C and HLE-R-C (Figure 5E). Downregulating the expression of PEX5 partially resensitized HepG2-R-C and HLE-R-C upon irradiation (Figure 5F). Thus, PEX5 desensitized HCC cells to radiation *in vitro*.

**Figure 6:**
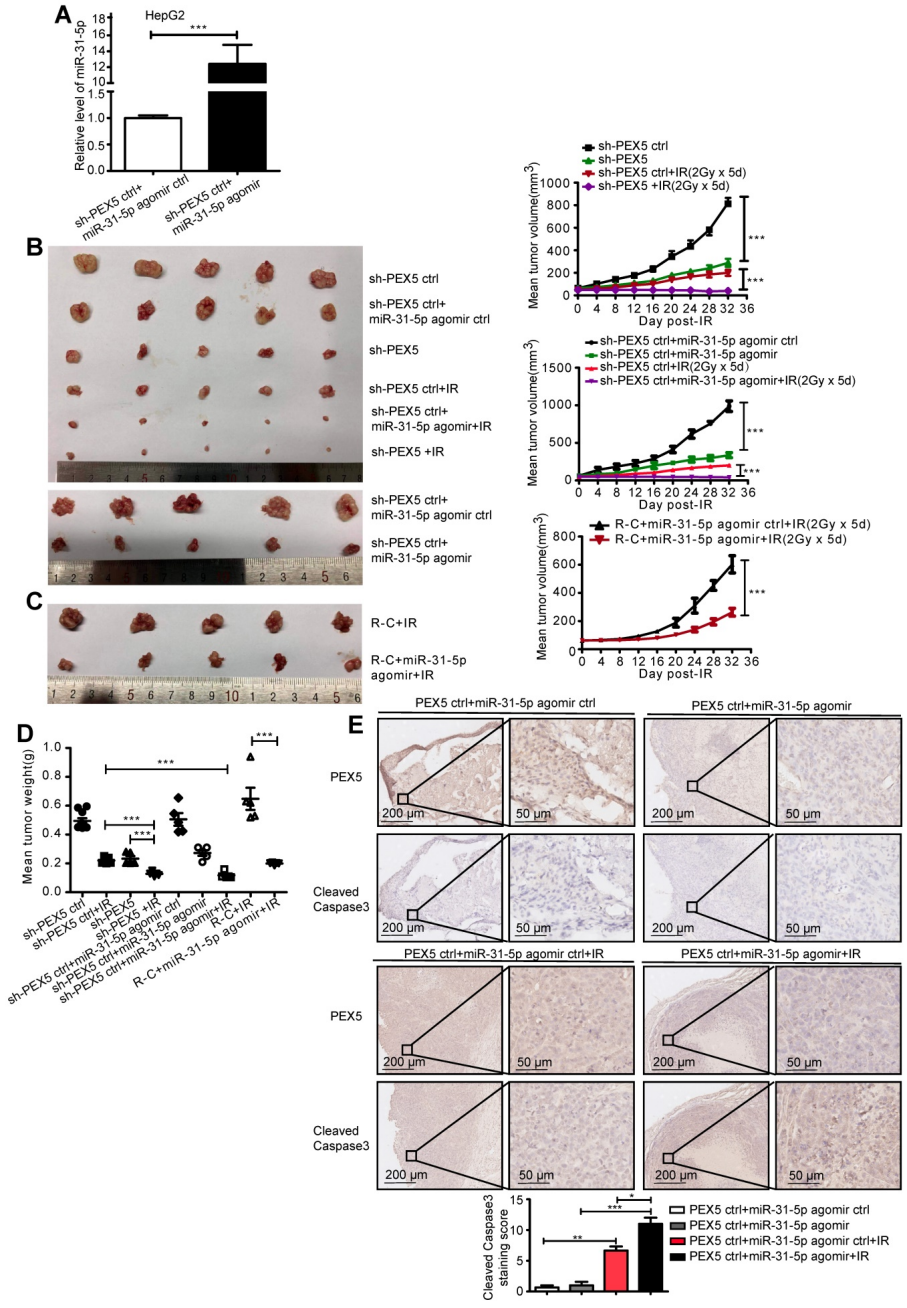
PEX5 desensitized HCC cells to radiation *in vivo,* but miR-31-5p reversed this effect. (A) The level of miR-31-5p was increased by the miR-31-5p agomir in HepG2 cells (***P < 0.001). (B-D) Volume and weight of HCC tumors with different levels of PEX5 and miR-31-5p expression after irradiation (n = 5, ***P < 0.001). (E) The protein levels of cleaved caspase-3 in irradiated HCC tumors with different levels of PEX5 and miR-31-5p expression were validated by IHC. Top: Representative images of cleaved caspase-3 and PEX5 staining in tumor tissues of different groups. Bottom: Statistical analysis results (*P < 0.05, **P < 0.01, ***P < 0.001). The results are representative of three independent experiments. Abbreviations: ctrl, control; sh-PEX5, shRNA-PEX5; GAPDH, glyceraldehyde 3-phosphate dehydrogenase; R-C, radiotherapy-resistant cells; IR, irradiation.

We validated the effects of PEX5 and miR-31-5p on HCC radiosensitivity *in vivo* by using a miR-31-5p agomir to elevate miR-31-5p expression levels (Figure 6A) and found that the growth of tumors treated with the combination of PEX5 knockdown and radiation was slower than that of tumors treated with radiation or PEX5 knockdown alone. The miR-31-5p agomir significantly decreased tumor growth after irradiation and reduced the radioresistance of HepG2-R-C (Figure 6B-C). A similar trend was observed for the tumor weights (Figure 6D). Upon irradiation, PEX5 knockdown or miR-31-5p upregulation increased cleaved caspase-3 levels (Figure 6E). Collectively, these results indicate that PEX5 increases the radioresistance of HCC cells *in vivo* and that this increase is reversible by expressing miR-31-5p.

### PEX5 promotes Wnt/β-catenin pathway signaling by stabilizing cytoplasmic β-catenin and assisting β-catenin nuclear translocation

Based on the results of the above phenotypic studies, we further explored the regulatory mechanism of PEX5 in the radiation response of HCC cells. The Wnt/β-catenin pathway plays an important role in regulating radioresistance in cancer 25. Interestingly, ONCOMINE analysis showed that positive CTNNB1 staining is correlated with high PEX5 protein expression in HCC (Figure S4A). In addition, Gene Expression Profiling Interactive Analysis (GEPIA) data showed that PEX5 levels are positively correlated with *CTNNB1* levels in HCC (Figure S4B). Therefore, we further explored the effects of miR-31-5p and/or PEX5 on β-catenin, the major downstream effector of the Wnt pathway.

**Figure 7:**
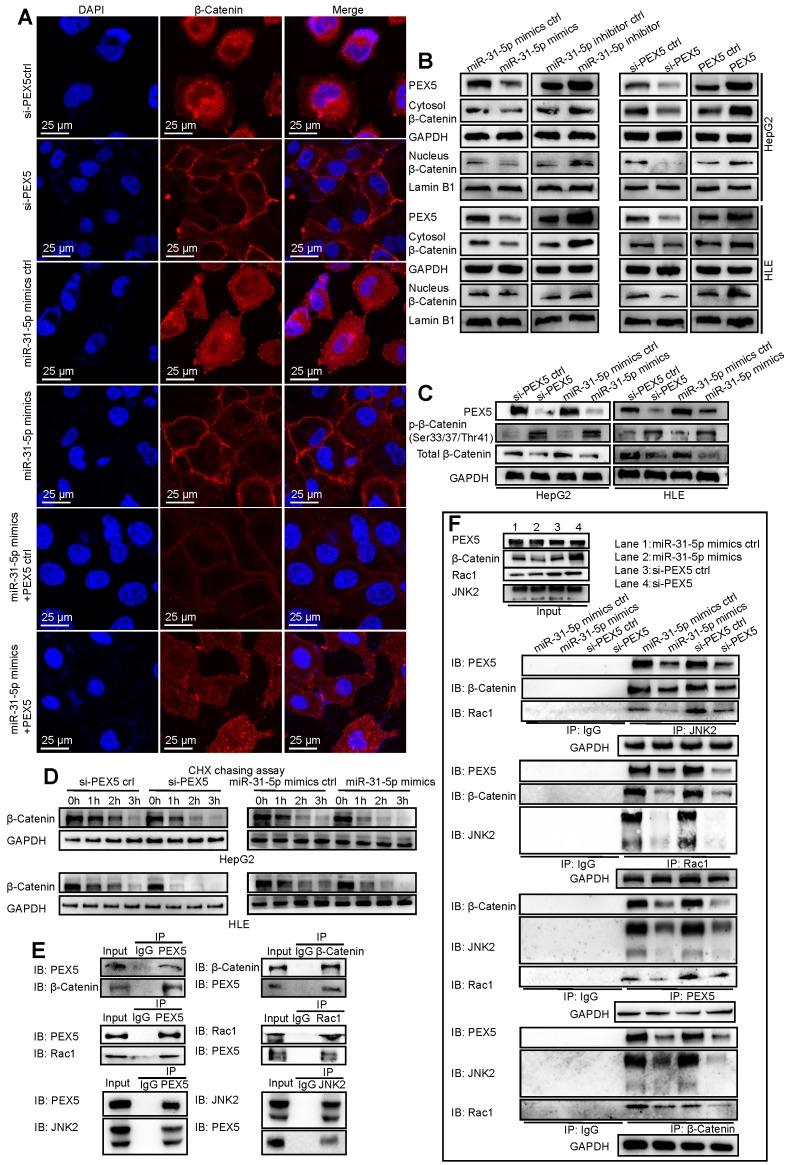
PEX5 stabilized β-catenin and promoted its nuclear translocation. After the levels of PEX5 and miR-31-5p in HepG2 cells were altered by transfection with the corresponding plasmids, the following experiments were performed. (A) The subcellular localization of β-catenin was validated by immunofluorescence. β-Catenin was labeled with Alexa Fluor®594 (red), and nuclei were labeled with DAPI (blue). Scale bar represents 25 μm. (B) The regulation of nuclear and total β-catenin levels by PEX5 was validated by WB. (C) The regulation of p-β-catenin and total β-catenin levels by PEX5 and miR-31-5p was demonstrated by WB. (D) The modulatory effects of PEX5 and miR-31-5p on the stabilization of β-catenin were validated by a CHX chase assay. (E) The interaction of PEX5 with Rac1, JNK2 and β-catenin was validated by Co-IP. (F) The effects of PEX5 and miR-31-5p on the interactions within the Rac1-JNK2-β-catenin complex were validated by Co-IP. All results are representative of three independent experiments. Abbreviations: ctrl, control; si-PEX5, siRNA-PEX5; GAPDH, glyceraldehyde 3-phosphate dehydrogenase.

As shown in Figure 7A, the staining intensity of both nuclear and total β-catenin was markedly decreased in HepG2 cells upon treatment with si-PEX5 or the miR-31-5p mimics. In addition, the reduction in the β-catenin staining intensity induced by the miR-31-5p mimics was reversed by ectopic expression of PEX5. In the HepG2 and HLE cell lines, the levels of nuclear and cytosolic β-catenin were decreased by the miR-31-5p mimics or si-PEX5, and treatment with the miR-31-5p inhibitor or ectopic expression of PEX5 produced the opposite effects (Figure 7B). However, the mRNA levels of β-catenin were not markedly changed by either ectopic expression or knockdown of miR-31-5p or PEX5 (Figure S4C), indicating that miR-31-5p and PEX5 may regulate β-catenin at the protein level. To this end, we observed increasing p-β-catenin levels accompanied by decreased decreased total β-catenin levels in both cell lines upon treatment with the miR-31-5p mimics or si-PEX5 (Figure 7C). Furthermore, the cycloheximide (CHX) chase assay showed that si-PEX5 and the miR-31-5p mimics facilitated destabilization of β-catenin (Figure 7D). In summary, miR-31-5p inhibited the increase in both the cytoplasmic and nuclear levels of β-catenin mediated by PEX5.

β-Catenin accumulates in the cytoplasm and subsequently translocates to the nucleus to activate the Wnt pathway. The Rac1-JNK2-β-catenin complex plays a critical role in the nuclear translocation of β-catenin 29. We found that PEX5 interacts with the Rac1-JNK2-β-catenin complex (Figure 7E) and that treatment with si-PEX5 or the miR-31-5p mimics impaired the formation of the complex (Figure 7F). Treatment with MG132, a proteasome inhibitor, inhibited the reduction in the β-catenin level mediated by si-PEX5 and the miR-31-5p mimics (Figure S4D). Although β-catenin levels were restored, the interaction among Rac1, JNK2 and β-catenin was decreased by treatment with si-PEX5 or the miR-31-5p mimics (Figure S4E), indicating a key role of PEX5 in the formation of the Rac1-JNK2-β-catenin complex. Upon si-PEX5 or miR-31-5p mimics treatment, formation of the complex was impaired, and β-catenin nuclear translocation was inhibited. However, the decrease in the nuclear β-catenin protein level induced by si-PEX5 and the miR-31-5p mimics were reversed by MG132, implying that PEX5 may not be the only factor mediating β-catenin nuclear translocation (Figure S4F). Collectively, these findings indicate that PEX5 plays a pivotal role in β-catenin nuclear translocation by facilitating the formation of the Rac1-JNK2-β-catenin complex.

Further, LF3, an antagonist of the β-catenin/TCF4 interaction, rescued PEX5 overexpression mediating the increase of radioresistance of HepG2 and HLE cell lines (Figure S4G). These results indicate that PEX5 promotes radioresistance via Wnt/β-catenin signaling.

### PEX5 promotes the proliferation of HCC cells *in vitro* and *in vivo*

Cells in different phases of the cell cycle possess distinct radiosensitivity. Activating the canonical Wnt pathway promotes radioresistance by increasing the population of S-phase cells, the majority of which are radioresistant 30. Furthermore, the accelerated reproliferation of cancer cells causes the increase in radioresistance 31. We thus hypothesized that PEX5-mediated activation of Wnt/β-catenin signaling increased HCC radioresistance via cell cycle redistribution and the promotion of proliferation.

**Figure 8:**
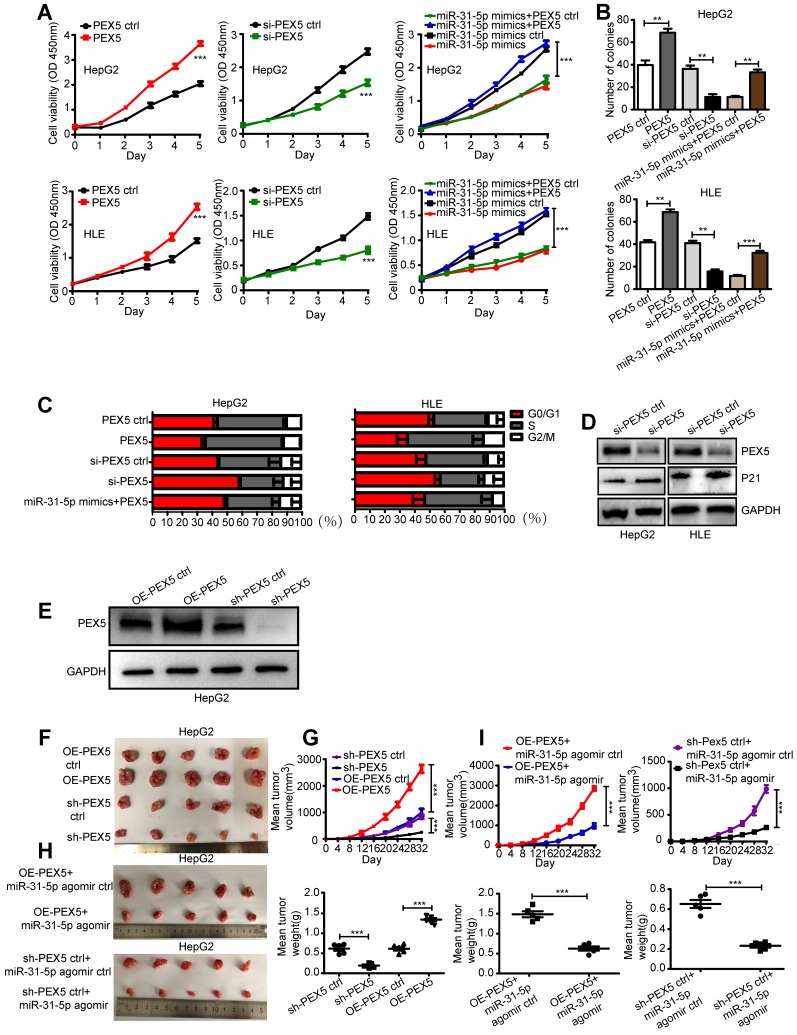
PEX5 promoted HCC cell proliferation* in vitro* and* in vivo*; however, miR-31-5p reversed this effect. (A) The effect of PEX5 on HCC cell growth was validated by a CCK8 assay (***P < 0.001). (B) Effect of PEX5 on HCC cell colony formation ability (**P < 0.01, ***P < 0.001). (C) In both cell lines, downregulation of PEX5 increased the proportion of cells in G1 phase, with a concomitant reduction in the proportion of cells in S phase and G2/M phase; however, upregulation of PEX5 inhibited this effect. The miR-31-5p mimics-induced cell cycle arrest in G1/S phase was rescued by PEX5. (D) si-PEX5 increased the protein level of p21^WAF1/Cip1^ in both cell lines. The results are representative of three independent experiments. (E) PEX5 protein level in OE-PEX5 and sh-PEX5 cells. (F-G) The volume and weight of HCC tumors with different levels of PEX5 expression (n = 5, ***P < 0.001). (H-I) Effect of miR-31-5p on the volume and weight of HCC tumors (n = 5, ***P < 0.001). Abbreviations: ctrl, control; si-PEX5, siRNA-PEX5; GAPDH, glyceraldehyde 3-phosphate dehydrogenase; sh-PEX5, shRNA-PEX5; OE-PEX5, PEX5 overexpression.

We showed that the miR-31-5p mimics reduced cell growth and colony numbers, whereas the miR-31-5p inhibitor increased cell proliferation and colony numbers in both cell lines (Figure S5A-B). In addition, overexpression of PEX5 significantly promoted but si-PEX5 suppressed cell proliferation. Ectopic expression of PEX5 abolished the inhibitory effect of the miR-31-5p mimics on cell viability (Figure 8A). Further, PEX5 increased the colony numbers, whereas si-PEX5 showed the opposite tendency. PEX5 rescued the miR-31-5p mimics-suppressed clonogenic capacity of both cell lines (Figure 8B). Transfection with either the miR-31-5p mimics or si-PEX5 led to G1/S cell cycle arrest, with a decreased proportion of cells in S phase and upregulated protein levels of p21^WAF1/Cip1^, which indicates cell cycle arrest. However, either miR-31-5p knockdown or PEX5 upregulation attenuated this trend in both cell types. In addition, the G1/S cell cycle arrest caused by the miR-31-5p mimics was rescued by PEX5 overexpression (Figure 8C-D, Figure S5C-D).

To investigate the effects of PEX5 and miR-31-5p on HCC growth *in vivo*, OE-PEX5, OE-PEX5 ctrl, sh-PEX5, and sh-PEX5 ctrl plasmids were transfected into HepG2 cells to establish stable overexpression or knockdown of PEX5 in cells (Figure 8E). These cells were further implanted into nude mice to study their tumor growth ability *in vivo*. PEX5 increased the tumor volume and weight (Figure 8F-G), whereas the miR-31-5p agomir inhibited PEX5-induced tumor growth *in vivo* (Figure 8H-I).

### PEX5 promotes epithelial-mesenchymal transition (EMT) and metastasis of HepG2 and HLE cells both *in vitro* and *in vivo*

EMT is recognized as a critical process in tumor migration and invasion. Recent studies have shown that EMT is associated with radioresistance 32. Activation of Wnt/β-catenin signaling induces EMT and promotes radioresistance in nasopharyngeal carcinoma 32. We thus hypothesized that PEX5-activated Wnt/β-catenin signaling increases HCC radioresistance by promoting EMT.

**Figure 9:**
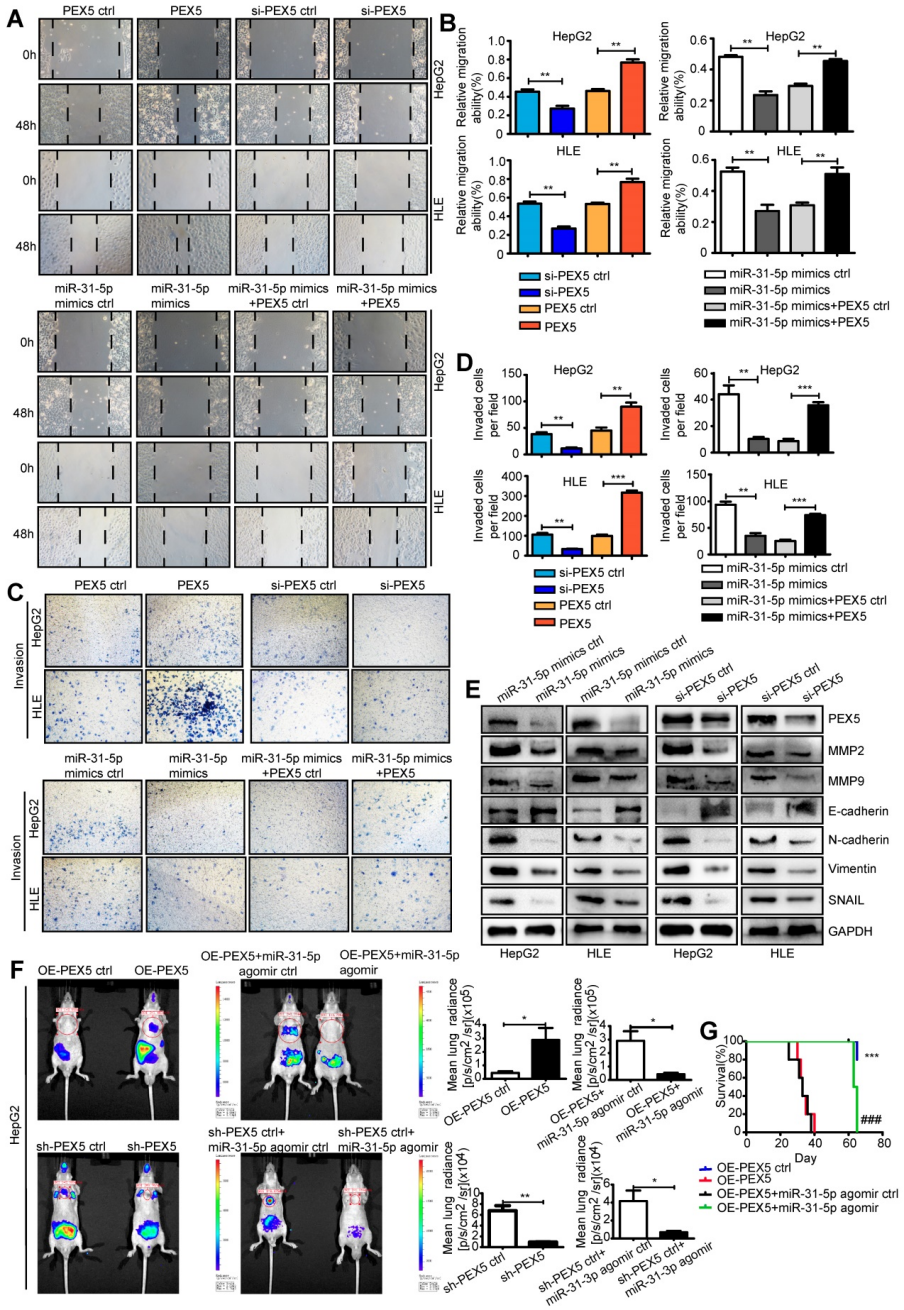
PEX5 promoted EMT and metastasis of HCC cells, but miR-31-5p inhibited these phenomena. (A-B) Wound-healing assay showing the effect of PEX5 on the migration ability of HCC cell lines. The results are representative of three independent experiments (**P < 0.01). (C-D) The effect of PEX5 on the invasion ability of HCC cell lines was evaluated with a Transwell assay. The results are representative of three independent experiments (**P < 0.01, ***P < 0.001). (E) The effects of miR-31-5p and PEX5 on the protein levels of MMP2, MMP9, E-cadherin, N-cadherin, Vimentin and SNAIL in both cell lines. The results are representative of three independent experiments. (F) *In vivo* imaging of lung metastasis in nude mice that received tail vein injection of luciferase-labeled HepG2 cells stably transduced with OE-PEX5, sh-PEX5 or the corresponding control plasmids. Further, the effect of miR-31-5p on lung metastasis was assessed with the miR-31-5p agomir or agomir ctrl. Luciferase signals are shown as dot plots (n = 5, *P < 0.05, **P < 0.01). (G) Survival analysis among groups with different miR-31-5p or PEX5 expression levels (n = 5; ***P < 0.001, OE-PEX5 group vs OE-PEX5 ctrl group; ###P < 0.001, OE-PEX5+miR-31-5p agomir group vs OE-PEX5+miR-31-5p agomir ctrl group). Abbreviations: ctrl, control; si-PEX5, siRNA-PEX5; GAPDH, glyceraldehyde 3-phosphate dehydrogenase; sh-PEX5, shRNA-PEX5; OE-PEX5, PEX5 overexpression.

We used a wound-healing assay to show that the miR-31-5p mimics attenuated cell migration (Figure S5E). The results of Transwell invasion assays showed that the miR-31-5p mimics also impaired the invasion abilities of HepG2 and HLE cells, whereas the miR-31-5p inhibitor reversed this phenotype (Figure S5F). Furthermore, in contrast to ectopic expression of PEX5, si-PEX5 suppressed the migration and invasion abilities of both cell lines. The inhibitory effects of the miR-31-5p mimics on the migration and invasion abilities were reversed by PEX5 (Figure 9A-D). Consistent with these findings, N-cadherin, vimentin, and SNAIL, which are involved in EMT, were downregulated, whereas E-cadherin was upregulated by the miR-31-5p mimics and si-PEX5. Moreover, the miR-31-5p mimics and si-PEX5 decreased the levels of MMP2 and MMP9, key molecules mediating tumor cell invasion (Figure 9E). Taken together, these results suggest that PEX5 promotes EMT of HepG2 and HLE cells to enhance their migration and invasion *in vitro*, whereas miR-31-5p suppresses this phenomenon.

To verify these effects on metastasis *in vivo*, OE-PEX5-, OE-PEX5 ctrl-, sh-PEX5-, and sh-PEX5 ctrl-transfected HepG2 cells were injected into mice via the tail vein. Bioluminescence signal intensity measurements showed that PEX5 overexpression promoted lung metastasis of HepG2 cells *in vivo*, whereas the miR-31-5p agomir repressed this tendency (Figure 9F). PEX5 overexpression significantly increased the incidence of lung metastasis, but the miR-31-5p agomir constrained it *in vivo* (Figure S5G, Table S4). As expected, PEX5 overexpression was associated with poor survival of mice. Furthermore, the PEX5-mediated worsening of the outcome was reversed by the miR-31-5p agomir (Figure 9G). Fewer Ki-67-, MMP2- and MMP9-positive cells were observed in tumors from mice treated with sh-PEX5 and the miR-31-5p agomir than in tumors from mice in the control groups (Figure S6A-H).

### PEX5 desensitizes HCC cells to radiation by activating the HR pathway and consuming excessive ROS

The direct effect of RT is to cause lethal DNA damage; however, whether PEX5 participates in the DNA damage repair process is unknown.

**Figure 10:**
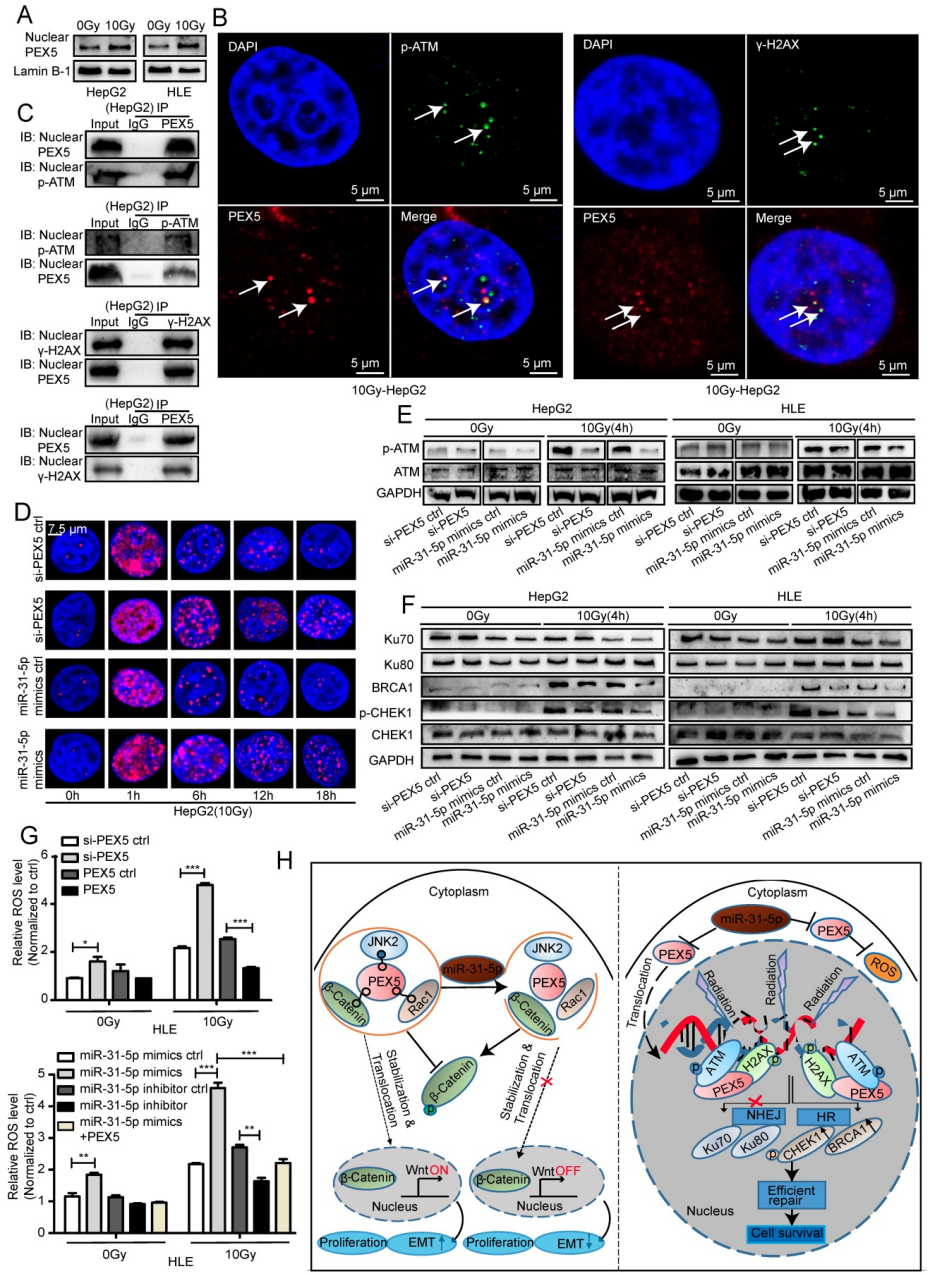
PEX5 activated the HR pathway and reduced radiation-induced ROS accumulation; however, miR-31-5p repressed this effect. (A) The nuclear PEX5 levels in both cell lines after irradiation were validated by WB. The results are representative of three independent experiments. (B) PEX5 was colocalized with p-ATM and γ-H2AX upon irradiation, as validated by immunofluorescence. The results are representative of three independent experiments. PEX5 was labeled with Alexa Fluor®594 (red), p-ATM or γ-H2AX with FITC 488 (green), and nuclei with DAPI (blue). (C) The interaction of PEX5 with p-ATM and γ-H2AX in the nucleus after irradiation was validated by Co-IP. The results are representative of three independent experiments. (D) Quantification of γ-H2AX foci in each nucleus at different time points after irradiation (n ≥ 50), as validated by immunofluorescence. γ-H2AX was labeled with Alexa Fluor®594 (red), and nuclei were labeled with DAPI (blue). (E-F) The effects of PEX5 and miR-31-5p on the levels of p-ATM, Ku70, Ku80, BRCA1, p-CHEK1 and CHEK1 at 4 h after irradiation were validated by WB. The results are representative of three independent experiments. (G) Effects of PEX5 and miR-31-5p on the ROS level in HLE cells with or without radiation exposure (*P < 0.05, **P < 0.01, ***P < 0.001). The results are representative of three independent experiments. (H) Schematic representation of miR-31-5p/PEX5 signaling in HCC. Abbreviations: ctrl, control; si-PEX5, siRNA-PEX5; GAPDH, glyceraldehyde 3-phosphate dehydrogenase; ROS, reactive oxygen species.

In the GEPIA dataset, the *PEX5* mRNA expression levels were correlated with the levels of DNA damage repair genes such as *CHEK1*, *BRCA1,* and *ATM*, suggesting that PEX5 may be involved in the DNA damage repair response (Figure S7A-C). Upon irradiation, the PEX5 intensity in the nucleus, along with the nuclear PEX5 protein level, was markedly increased (Figure S7D, Figure 10A). As shown in Figure 10B-C and Figure S7E, upon irradiation, PEX5 colocalized with p-ATM and γ-H2AX foci. In addition, the coimmunoprecipitation (Co-IP) results showed that PEX5 interacted with p-ATM and γ-H2AX in the nucleus after irradiation. The number of γ-H2AX foci is reported to be proportional to the amount of DNA strand breaks 33. Therefore, we then performed qualitative and quantitative measurements of DNA damage by immunofluorescence staining of γ-H2AX foci. Treatment with the miR-31-5p mimics or si-PEX5 increased the average number of γ-H2AX foci at 6, 12, and 18 h post irradiation, indicating that both the miR-31-5p mimics and si-PEX5 inhibited the DNA repair process (Figure 10D, Figure S7F). Consistent with this result, the γ-H2AX level was increased by the miR-31-5p mimics and si-PEX5 in both cell lines at 12 h after irradiation (Figure S7G). In addition, the p-ATM level was decreased by si-PEX5 and the miR-31-5p mimics at 4 h after irradiation (Figure 10E). Notably, upon irradiation, the levels of HR pathway components, such as BRCA1 and p-CHEK1, were decreased by the miR-31-5p mimics or si-PEX5, whereas the levels of the proteins involved in the NHEJ pathway, such as Ku70 and Ku80, were not changed significantly (Figure 10F). These findings suggest that PEX5 protects cells from radiation by promoting HR pathway but not NHEJ pathway activity. Although excess ROS generated by ionizing radiation are well recognized as mediators of DNA damage 34, radiation-induced formation of excessive ROS was reduced by PEX5 overexpression and miR-31-5p knockdown (Figure 10G).

## Discussion

Our findings in this study demonstrated that the miR-31-5p level is reduced in HCC and modulates tumor radioresistance through PEX5-mediated regulation of the Wnt and HR signaling pathways (Figure 10H). Consistent with our current observations, several studies have demonstrated reduced expression of miR-31-5p in HCC 35, 36 and its close association with poor clinical outcome 37, 38. Recently, studies have implied that miR-31-5p plays a radiation-induced proapoptotic role in esophageal adenocarcinoma cells 11 and mouse intestinal stem cells 39, via which various DNA damage repair genes are directly or indirectly suppressed. Consistent with this observation, our previously published work showed that miR-31-5p is induced in colorectal cancer cells in response to radiation injury 40. Thus, the functions of miR-31-5p in inhibiting radioresistance are common to different tumor tissues.

Here, a striking finding was that miR-31-5p suppressed the canonical Wnt/β-catenin signaling pathway by targeting PEX5. The Wnt/β-catenin signaling pathway is usually hyperactivated in liver cancers 41, consistent with our observations of decreased miR-31-5p and increased PEX5 levels in HCC. In many types of cells, activation of the Wnt/β-catenin signaling pathway contributes to radioresistance via intricate mechanisms 42. Notably, this prosurvival signaling pathway influences radioresistance through both ROS and the DNA damage response. The absence of β-catenin decreases catalase expression and elevates ROS levels upon irradiation 43. Activation of the Wnt/β-catenin pathway induces the DNA damage response in breast epithelial cells, leading to elevated p-Chk2, γ-H2AX, pRb, p16 and p53 levels 44. In addition, upon irradiation, β-catenin upregulates the expression of MRE11, which participates in DNA damage recognition 45. C-myc, a β-catenin target gene, promotes the transcription of HR-related RAD51 in the DU145 and H1299 cell lines 46. Furthermore, activated Wnt/β-catenin signaling also desensitizes cancer cells to radiation via cell cycle redistribution, acceleration of proliferation, inhibition of apoptosis, and promotion of cell invasion and tumor cell differentiation 14, 47, 48. Furthermore, activated Wnt signaling contributes to EMT, which increases the radioresistance of cancer cells 49-51. These findings indicate that the miR-31-PEX5-Wnt/β-catenin axis is an important contributor to tumor radioresistance in HCC.

Stabilization and nuclear translocation of β-catenin are key steps in canonical Wnt signaling 52. Inactive β-catenin is phosphorylated at Ser33/37/Thr41 by a degradation complex including GSK3b, and immediately undergoes proteasomal degradation. Activation of Wnt signaling blocks the degradation complex, leading to stabilization, cytosolic accumulation, and subsequent nuclear translocation of β-catenin 53. However, the exact β-catenin shuttle mechanism is incompletely understood 54.

Our current findings demonstrated that both miR-31-5p and si-PEX5 significantly decrease the protein level of β-catenin in both the cytosol and nucleus, accompanied by an increase in p-β-catenin (Ser33/37/Thr41), indicating the importance of miR-31 and PEX5 in regulating β-catenin degradation. These effects were abolished by the proteasome inhibitor MG132, indicating that PEX5 may support the accumulation of β-catenin by inhibiting its degradation. In addition, previous reports have shown that the Rac1-JNK2-β-catenin complex is an important participant in this process 29, 55, 56. Consistent with this observation, we revealed that PEX5 facilitates the formation of the Rac1-JNK2-β-catenin complex, suggesting that PEX5 may also be involved in nuclear translocation of β-catenin via this complex.

To our knowledge, most previous studies on PEX5 have focused on PEX5-mediated protein import pathways. PEX5 can recognize and bind cytosolic soluble proteins with surface-exposed PTS1 and transport them into the peroxisome matrix 57. However, our findings reveal a novel function of PEX5 in facilitating β-catenin nuclear translocation. This finding suggests that PEX5 participates in diverse transport mechanisms that are not limited to its identification of PTS1. We report a novel discovery that PEX5 potentially stabilizes cytoplasmic β-catenin by participating in the Rac1-JNK2-β-catenin complex to activate the Wnt signaling pathway, adding a novel mechanism to the complicated regulatory network of the Wnt/β-catenin pathway.

HR is an error-free DNA damage repair pathway 58 and requires a double-stranded sister chromatid as a homologous template. The repair process is mediated by the MRE11/RAD50/NBS1 complex along with RPA, RAD51, XRCC2, XRCC3 and BRCA1/2 59-61. In this study, we found that knockdown of PEX5 resulted in impairment of the DNA damage response, as evidenced by a decrease in p-ATM levels and retention of γ-H2AX in the nucleus, and identified that PEX5 is relocated to the nucleus to activate the HR pathway. These results suggest that PEX5 functions as a novel regulator of the HR pathway. We thus speculated that PEX5 may function as a DNA damage sensor under radiation stress. The regulatory mechanism underlying PEX5-mediated HR activation merits further investigation. This finding was consistent with our previous observation that HCC patients with high nuclear PEX5 levels had poor clinical outcomes. Based on these previous findings and our current data, we propose that PEX5 desensitizes HCC cells to radiation by scavenging excessive ROS and promoting HR pathway activity to protect cells from radiation stress.

## Conclusions

We innovatively demonstrated that miR-31-5p targets PEX5 to inhibit Wnt signaling by decreasing the stabilization and nuclear translocation of β-catenin and suppresses HR signaling by decreasing BRCA1 and p-CHEK1 levels upon irradiation. Thus, our data elucidated a novel mechanism connecting PEX5, the Wnt/β-catenin pathway, the HR pathway, and radioresistance and provided preclinical evidence to suggest PEX5 and miR-31-5p as therapeutic targets in HCC. However, the details of this mechanism should be further explored. Large clinical trials that investigate the correlation among miR-31-5p, PEX5, and clinical features are required.

## Supplementary Material

Supplementary materials and methods, figures, and tables.

## Abbreviations

ctrl: Control
miR-31: MicroRNA-31
DMEM: Dulbecco's modified Eagle's medium
FBS: Fetal bovine serum
ROS: Reactive oxygen species
R-C: Radiotherapy-resistant cells
IF: Immunofluorescence staining
IHC: Immunohistochemistry
ISH: *In situ* hybridization
TCGA: The Cancer Genome Atlas
GAPDH: Glyceraldehyde 3-phosphate dehydrogenase
qRT-PCR: Quantitative real-time polymerase chain reaction
HE: Hematoxylin and eosin-stained
Co-IP: Coimmunoprecipitation
HR: Homologous recombination
NHEJ: Nonhomologous end joining
RT: Radiation therapy
CCK8: Cell Counting Kit-8
WB: Western blotting
T: Tumor tissues
N: Normal tissues
mut: mutant
si-PEX5: SiRNA-PEX5
WT: Wild-type
sh-PEX5: ShRNA-PEX5
DAPI: 4',6-Diamidino-2-phenylindole
CHX: Cycloheximide
IP: Immunoprecipitation
IB: Immunoblotting
OD: Optical density
OE-PEX5: PEX5 overexpression
