# Supplementary material for: PEX5, a novel target of microRNA-31-5p, increases radioresistance in hepatocellular carcinoma by activating Wnt/β-catenin signaling and homologous recombination
Source: Theranostics. 2020 Apr 6;10(12):5322–40. doi: 10.7150/thno.42371 (PMC7196300; doi:10.7150/thno.42371)
Supplement: Supplementary file 1 — Supplementary materials and methods, figures, and tables. [file thnov10p5322s1.pdf]

PEX5, a novel target of microRNA-31-5p, increases radioresistance in hepatocellular carcinoma by activating Wnt/β-catenin signaling and homologous recombination.

Jie Wen[1], Kai Xiong[1], Abudureyimujiang Aili[1], Hao Wang[1], Yuequan Zhu[2], Zhengquan Yu[3], Xueyan Yao[4], Ping Jiang[1], Lixiang Xue[1#], Junjie Wang[1#]

Table of contents

Supplementary materials and methods……………………..………..…2

Supplementary Table 1……………………………………………………19

Supplementary Table 2……………………………………………………20

Supplementary Table 3……………………………………………………21

Supplementary Table 4……………………………………………………22

Supplementary Table 5……………………………………………………22

Supplementary Table 6……………………………………………………23

Supplementary Fig. 1………………………………………………………25

Supplementary Fig. 2………………………………………………………27

Supplementary Fig. 3………………………………………………………29

Supplementary Fig. 4………………………………………………………31

Supplementary Fig. 5………………………………………………………33

Supplementary Fig. 6………………………………………………………35

Supplementary Fig. 7………………………………………………………37

Supplementary Fig. 8………………………………………………………39

Supplementary references……………………………………………....41

Supplementary materials and methods

Cell culture

The human HCC cell lines HepG2, HLE and 293T were obtained from Shanghai Cell Bank (Shanghai, China) and cultured in Dulbecco's Modified Eagle Medium (DMEM, Corning) supplemented with 10% fetal bovine serum (FBS, Excell Bio, FSP500) and 1% antibiotic-antimycotic at 37 °C in a humidified atmosphere of 5% CO2 and 95% air.

Extraction of total RNA and RT-qPCR assay

According to the product manual, TRIzol reagent (Invitrogen) was applied to isolate total RNA from the tissue samples and cell samples according to the manufacture's introduction. Then, NanoDrop (Thermo Fisher Scientific, 2000C) was used to measure the concentration of RNAs of different samples. After that, for detection of miRNA level, the miRcute Plus miRNA First-Strand cDNA Kit (TIANGEN, China) was used to synthesize the cDNA for miRNA and the qRT-PCR reactions were performed with miRcute Plus miRNA qPCR Kit (SYBR Green) (TIANGEN, China) and monitored in real-time by QuantStudio 5 (Thermo Fisher Scientific). The internal control was U6. The miRNA cDNA synthesis program was described as follows: 42 ℃ (60 min) then 95 ℃ (3 min). The miRNA detection program was described as follows: 95 ℃ (15 min) for one cycle, and followed by 45 cycle (20 s at 95°C, 34 s at 60°C). The miRNA qPCR reaction mixture volume was 20 μL and included 10 μL miRNA reaction buffer, 2 μL miRNA enzyme mix and 2 μg RNA. The miRNA qPCR detection mixture volume was 20 μL and included 10 μL miRcute plus miRNA premix, 0.4 μL forward primer, 0.4 μL reverse primer, 1 μL miRNA first-strand cDNA and 1.6 μL ROX.

For detection of mRNA, FastKing gDNA Dispelling RT SuperMix (TIANGEN, China) was used to synthesize the cDNA for mRNA, Talent qPCR PreMix (SYBR Green)

(TIANGEN, China) was used to detect the mRNA expression. The internal control was GAPDH. The mRNA cDNA synthesis program was described as follows: 42 ℃ (15 min) then 95 ℃ (3 min). The mRNA detection program was described as follows: 95 ℃ (15 min) for one cycle, and followed by 40 cycle (10 s at 95°C, 32 s at 66°C), 10 s at 95 ℃. The mRNA synthesis program includes gDNA elimination (2 μL gDNA buffer, 2 μg RNA) and reverse transcription reaction (2 μL fast RT buffer, 1 μL enzyme mix, 2 μL primer mix). The mRNA qPCR detection mixture volume was 20 μL and included 10 μL superreal premix plus, 0.6 μL forward primer, 0.6 μL reverse primer, 1 μL cDNA and 0.4 μL ROX. The $2^{-\Delta\Delta CT}$ means were applied to calculate the value of the expression. qRT-PCR analysis was performed using QuantStudio 5. The detail primer sequences were listed in Supplementary Table 5.

Western blotting (WB) analysis and immunoprecipitation assay

The whole-cell lysate and tissue lysate was extracted with RIPA lysis buffer (50mM Tris [pH 7.4], 150mM NaCl, 1% Triton X-100, 1% sodium deoxycholate, 0.1% SDS, sodium orthovanadate, sodium fluoride, EDTA, from Beyotime Company). Then BCA assay was used to measure the protein concentration. Afterwards, 30 μg protein was loaded onto sodium dodecyl sulfate-polyacrylamide gel (SDS-PAGE) to separate. After that, proteins on the gels were transferred to polyvinylidene fluoride (PVDF, 0.22um, Millipore Company) membrane, and the transferring time was based on the protein molecular weight. Then, the membranes were incubated with primary antibodies (at the appropriate dilution according to the supplementary table 6) in 6 ml primary antibody dilution buffer with gentle agitation overnight at 4°C. Followed that, the membranes were incubated with Anti-rabbit/mouse IgG, HRP-linked antibody (1:2000, Beyotime Company) in 10 ml secondary antibody dilution buffer with gentle agitation for 2 hours at room temperature. After washing three times for 5 min each with 15 mL of Tris

Buffered Saline with 0.1% Tween® 20 (0.1% TBST), the membranes were exposed to get the blot images with 1X SignalFire™ ECL Reagent (Millipore Company).

For Co-Immunoprecipitation (Co-IP) experiment, cultured cells were lysed with moderate RIPA lysis buffer (50mM Tris [pH7.4], 150mM NaCl, 1% NP-40, 0.25% sodium deoxycholate, sodium orthovanadate, sodium fluorid, EDTA, Beyotime Company) and extracted proteins. Then, concentration of protein was detected by BCA assay. 500 μg protein diluted with 0.25 mL buffer was incubated with 4 μg antibody or normal rabbit/mouse immunoglobulin G (IgG, Proteintech Company) with gentle rolling over night at 4°C. After that, Protein A/G Plus-Agarose (Santa Cruz Biotechnology) was added into the protein samples to be incubated for 2 h at room temperature. Then, the agarose beads were collected to perform WB.

For the nuclear-cytosol proteins detection, cultured cells were harvested and lysed with Nuclear-Cytosol Extraction Kit (from APPLYGEN Company) according to the manufacture's introduction. All buffers should be kept on ice. The total cell protein was lysed with 300μL Cytosol Extraction Buffer A (CEB-A), then, vortexed for 30 sec, incubated on ice for 10 min and vortexed for 30 sec every 5 min for 3 times. Then, the sample was centrifuged at 1,000g 4°C for 10 min, and the supernatant was transferred to a new tube and further centrifuged at 12,000g for 5 min at 4°C. The supernatant was cytosol extract, and transferred to a new tube. The pellet contained crude nuclei. To remove those membrane components, the crude nuclei pellet was re-suspended in 100 μL CEB-A, added with 5 μL CEB, vortexed for 10 sec, and incubated on ice for 1 min. The tube was centrifuged for 5 min at 1,000g. Nuclei pellet was collected and the supernatant was discarded. After washing the nuclei pellet with 100 μL CEB-A, spinning for 5 min at 1,000g and discarding the supernatant, 100 μL cold Nuclear Extraction Buffer (NEB) was added into the samples, which were incubated on ice for

30 min. After centrifugation at 12,000g for 5 min at 4°C, the supernatant fraction containing the protein extracted from nucleus was transferred to a clean tube. The oncentration of protein was detected using the BCA assay. Then, the samples were analyzed by WB.

To detect the regulation of PEX5 and miR-31-5p on the protein level of Caspase-3 plasmids after radiation, HepG2 and HLE cell lines were transfected with miR-31-5p mimics, miR-31-5p mimics ctrl, si-PEX5 and si-PEX5 ctrl for 48 h separately. Next, both of the cells were exposed to radiation of 10Gy and cultured for 48 h. Then, both of the cells were collected for WB.

The detail information of antibodies was listed in Supplementary Table 6.

Immunocytochemistry (IHC)

Tissue samples were removed from nude mice and fixed in paraformaldehyde (4%) for 24 hours, and then dehydrated and embedded to make sections (5um, thick). Tissue sections were deparaffinized and hydrated, and then put into the heating sodium citrate buffer (10 mmol/L, pH 6.0) at 95°C for 15 minutes for antigen retrieval. Then, the sections were incubated with 3% H2O2 for 5-10 minutes at room temperature to eliminate endogenous peroxidase activity and washed with phosphate buffer saline (PBS), every 2 min for 3 times. Unspecific binding was blocked by incubating with 5~10% goat serum for 10 min at room temperature. Subsequently, primary antibodies were applied to sections and incubated overnight at 4°C, followed by the PV-9000 2-step plus Poly-HRP anti-Mouse/Rabbit IgG Detection system (Zhong Shan Jin Qiao, China). Then, the sections were further developed with DAB solution. Counter-staining was performed with hematoxylin and embedded with neutral balsam. Images were captured with NDP (Nano Zoomer Digital Pathology) scan system.

The detail information of antibodies was listed in supplementary table 6.

In situ hybridization

All of the reagents and containers were pre-treated with DEPC water. The 5 μm-thick paraffin sections were dewaxed with xylene and alcohol, then, deproteinized with 10 μL/mL proteinase K (Amresco), 0.2% glycine, 4% paraformaldehyde. After prehybridization in prehybridization solution at 60 °C for 2 hours, the sections were hybridized with Digoxin-labeled miRCURYLNA probe (EXIQON) for 16h at 60 °C. After washing, the sections were incubated with the anti-DIG-HRP at 37 ° C for 45 min, then, further developed with DAB solution. After counter-staining with hematoxylin and embedding with neutral balsam, images were captured with NDP system.

Wound healing assay

After transfection for 24 h, HepG2 and HLE cell lines were seeded into 6-well plates (about 6×10^5 cells per well). When cells growth reached 95-100% confluence, 200-μL pipette tip was used to scratch the cell. Then, the wells were washed with PBS thrice, and the medium was change to serum-free medium. The images of this wound were captured with microscope at 0 and 48 h (Nikon, Japan, L300N/300ND).

Invasion Assay

For detection of cell invasion ability *in vitro*, the transwell chamber inserts (8 mm pore size, Corning) and the Matrigel (Corning, BD Biocoat) were used. Matrigel (200 μL) was coated onto the upper compartment of insert for 4 h at 37 °C. After transfection for 24 h. HepG2 and HLE cell lines were harvested. Then, about 2.5×10^4 cells were resuspended in 200 μL medium with 1% FBS and seeded into the upper chamber after cell transfection. Medium with 10% FBS was added into the bottom chamber. After 24 h, the non-invading cells were removed from top chambers, and the invading cells in the lower chambers were fixed with 4% paraformaldehyde at room temperature for 10 min, then, stained with 0.1% methylene blue for 30 min and washed three times with

PBS. Afterwards, the images of cells were photographed with microscope (Nikon, Japan, L300N/300ND).

CCK8 assay

The HepG2 and HLE cells were seeded in 96-well plates with a density of 0.4×10^4 cells/well after transfection. And CCK-8 kit (Dojindo Laboratories, Japan) was used to detect the cell viability. 100 μL medium containing 10μL CCK8 was added into the each well at 0 h, 24 h, 48 h, 72 h, 96 h and 120 h after transfection. Then the optical density (OD) at 450 nm was measured with a multilabel plate reader (Thermo Fisher Scientific, US6111636) after 2 hours of incubation.

Cell transfection

miR-31-5p mimics, miR-31-5p mimics ctrl, miR-31-5p inhibitor, miR-31-5p inhibitor ctrl were purchased from Ribobio Biotech (Guangzhou, China). PcDNA-PEX5 (PEX5), PEX5 ctrl, PEX5 small interfering RNA (si-PEX5), si-PEX5 ctrl plasmids were purchased from Vigene Biosciences (Shandong, China). HepG2 and HLE cells were seeded into the 6-well plates at a density of 3×10^5 cells/well and left overnight. When they reached 50-60% confluence, the lipofectamine 3000 (Invitrogen) was used to perform cell transfection with 4 μg plasmids/5 μg RNA and 10 μL lipofectamine reagent per /well according to manufacturer's instruction manual. Finally, after 48 h, transfection efficiency was validated by qRT-PCR or WB.

Cell cycle assay

After transfection for 48 h, HepG2 and HLE cells were harvested with trypsin and washed three-times with PBS. After centrifugation, the cells were fixed with 3 mL of pre-cold 70% ethanol over 18 h. Next, the fixed cells were centrifuged, then incubated with RNase A (100 μg/mL) for 30 min at 37 °C. Followed, 7-amino-actinomycin D (7AAD) (Biolegend) was used to detect the cell cycle according to the manufacturer's

instruction. At last, the cell cycle was measured by the flow cytometry (BD FACS Calibur) and the cell cycle phase was calculated Modifit 3.0 software.

Dual luciferase report assay

The 3'UTR of PEX5 containing miR-31-5p binding site was amplified and cloned to pGL3 vector to obtain the pGL3-PEX5 vector. Moreover, the binding site of PEX5 was mutated to obtain the pGL3-PEX5 Mut vector, containing four nucleotide substitutions. And the pGL3-NC (negative control) was provided by TOLO BIOTECHNOLOGY Company (Shanghai, China). Then, pGL3-PEX5 vector, pGL3-PEX5 Mut vector or pGL3-NC vector was co-transfected with miR-31-5p mimics, miR-31-5p mimics ctrl, miR-31-5p inhibitor or miR-31-5p inhibitor ctrl and Renilla luciferase vector (Promega) in 293T cells with Lipofectamine3000 (Invitrogen). After 48 h, the cells were collected and the Dual-Luciferase Reporter Assay System (Promega) was used to determine luciferase activity values following the product instruction manual.

Colony formation assay

Colony formation assay was used to detect the radiation sensitivity of HepG2 and HLE cells with different level of miR-31-5p and PEX5. Different numbers of HepG2 and HLE cells transfected with plasmids for 48 h, were seeded into 6-well plates depending on the doses of radiation: 0Gy (100 cells/well), 2Gy (200 cells/well), 4Gy (800 cells/well), 6Gy (1600 cells/well), 8Gy (3200 cells/well) and 10Gy (6400 cells/well). And, the transfected cells were subjected to radiation at the above indicated doses. Then, the cells were cultured for 14 days to form colonies. Next, the colonies were fixed with methanol/acetic acid and stained with stained with methylene blue. Those colonies (≥50 cells) were then counted. The experimental data was fitted to the single-hit multi-target model to make the survival curves of different groups.

Immunofluorescence staining (IF)

For detection of the intracellular distribution of β-catenin, HepG2 cells were transfected with miR-31-5p mimics, miR-31-5p mimics ctrl, si-PEX5 and si-PEX5 ctrl plasmids for 24 h. And the rescued experiment was conducted by co-transfecting PEX5 plasmid and miR-31-5p into HepG2 cells for 24 h. Then, the transfected cells (about 1 x 10^5 cells) were seeded on 35 mm confocal dish (Corning) and left overnight. Next, the cells were fixed in 2% paraformaldehyde at room temperature for 15 min and treated with 0.1% Triton-100 diluted by cold PBS for 10min, then, washed with cold PBS for three times and blocked with normal goat serum for 30 min at 37 °C. Next, β-catenin primary antibody(1:100) was applied at 4°C overnight, followed by addition of Anti-rabbit IgG (H+L), F(ab')2 Fragment (Alexa Fluor®594) conjugated secondary antibody for 30 min at 37°C and DAPI for 15 min at room temperature. Last, after washing with cold PBS for three times, the cells were photographed by using a STED confocal microscopy (Leica SP8 STED).

For observation of the foci of γ-H2AX, HepG2 cells were transfected with miR-31-5p mimics, miR-31-5p mimics ctrl, si-PEX5 and si-PEX5 ctrl plasmids for 24 h. And the rescued experiment was conducted by co-transfecting PEX5 plasmid and miR-31-5p into HepG2 cells for 24 h. Then, the transfected cells (about 1 x 10^5 cells) were seeded on 35 mm confocal dish (Corning) and left overnight and then exposed to radiation of 10Gy. Then, at different time points after radiation (0 h, 1 h, 6 h, 12 h and 18 h) the cells were fixed in 2% paraformaldehyde at room temperature for 15min and treated 0.1% Triton-100 for 10 min, afterwards, washed with PBS three times and blocked with normal goat serum for 30 min at 37 °C. Next, γ-H2AX primary antibody (1:100) was incubated at 4°C overnight, followed by addition of Anti-rabbit IgG (Alexa Fluor®594) Conjugate secondary antibodies for 30 min at 37 °C and DAPI for 15 min at room temperature. Last, after washing with cold PBS for three times, the cells were

photographed by using a STED confocal microscopy (Leica SP8 STED).

For validation of the co-localization of PEX5 and P-ATM or γ-H2AX foci, HepG2 and HLE cells (about 1 x 10^5 cells) were seeded on 35 mm confocal dish (Corning), and left overnight, followed by radiation of 10Gy. After 4 h, the cells were fixed for 15 min with 2% paraformaldehyde at room temperature and treated with 0.1% Triton-100 for 15 min, then, washed with PBS for three times and blocked with normal goat serum for 30 min at 37 °C. Next, PEX5 primary antibody (1:200) was applied at 4°C overnight with P-ATM antibody (1:200) or γ-H2AX antibody (1:100), followed by addition of Anti-rabbit IgG (Alexa Fluor®594) and Anti-mouse IgG (FITC-488) for 30 min at 37 °C and DAPI for 15 min at room temperature. Last, after washing with cold PBS for three times, the cells were photographed by using a STED confocal microscopy (Leica SP8 STED).

JC-1 assay

JC-1 (5,5′,6,6′-tetrachloro-1,1′,3,3′-tetraethylbenzimidazolocarbocyanine iodide) can be used as a probe for cytofluorometry analysis to detect the mitochondrial transmembrane potential (MTP) in living cells [1]. The loss of mitochondrial membrane potential ($\Delta\psi m$) could be observed in apoptotic cells, indicating a transformation of J-aggregates (polarized mitochondria) into JC-1 monomers (depolarized mitochondria) [2]. As measured by flow cytometry, in the apoptotic cells, a decreased red fluorescence and a gain of green fluorescence could be detected [3]. A mitochondrial membrane potential assay kit with JC-1 was purchased from Beyotime Biotechnology company. To detect the effects of miR-31-5p and PEX5 on mitochondrial membrane potential, HepG2 and HLE cell lines were transfected with miR-31-5p mimics, miR-31-5p mimics ctrl, si-PEX5 and si-PEX5 ctrl plasmids for 48 h. Then the cells were collected with trypsin, washed with cold PBS for three times and loaded with JC-1 probes (5 x 10^5 cells/0.5 mL JC-1) in dark for 20 min at 37 ℃ according to the manufacturer's

instruction. Next, mitochondrial membrane potential was measured using the flow cytometry (BD FACS Calibur). To detect the effects of miR-31-5p and PEX5 on mitochondrial membrane potential after radiation, HepG2 and HLE cells were transfected with different plasmids for 48 h, then the cells were exposed to radiation of 10Gy and cultured for 48h. Next, the cells were collected to be loaded with JC-1 probes (5×10^5 cells/0.5 mL JC-1) in dark for 20 min at 37 ℃ according to the manufacturer's instruction. Mitochondrial membrane potential was measured using the flow cytometry (BD FACS Calibur). Quantification was carried out by counting the ratio of red to green signals.

Radiotherapy resistant cell (R-C)

To establish the radiation resistance cell lines for HepG2 and HLE, sequential radiation was applied. HepG2 and HLE cell lines were exposed to radiation of 2Gy. Then, the irradiated cell lines were cultured until they were able to repair damages induced by radiation and continue proliferation. Then, the cells were exposed to further radiation of 2Gy and cultured until they could continue proliferation after exposed to radiation each time. Next, irradiation of 4Gy twice, 6Gy twice, 8Gy twice and 10Gy twice were applied to the cells. Cell should be cultured for a period of time to repair the damages caused by irradiation until they could continue proliferation. Then, the cells were exposed to a new round of radiation of higher dose. The process took about half a year.

Biological information analysis

To predict target genes of miR-31-5p in HCC, the biological information analysis was applied. Firstly, the common miRNA databases, including miRWalk, Micro T4, miRanda, miRBridge, miRDB, miRMap, Target scan and PICTAR2 were used to make a common prediction of target genes. And as for the low expression of miR-31-5p in HCC, ONCOMINE database and UALCAN database were used to predict the common

high expression genes in HCC. Then the shared genes: *KHDRBS3*, *TACC2* and *PEX5*, were elected to be the commonly predicted target genes of miR-31-5p in HCC.

Stable knockdown and overexpression of PEX5

PEX5 pLent-EF1A-Luciferase-Puro (OE-PEX5) and ctrl (OE-PEX5 ctrl), shPEX5 pLent-U6-Luciferase-Puro (sh-PEX5) and ctrl (sh-PEX5 ctrl) were bought from Vigene Biosciences (Shandong, China). And, HepG2 cells were transfected with the specific lentiviral vectors according to the products manual for 72h. Puromycin (3ug/ml) was used for three days to kill those cells which were failed to be transfected. The rest of the cells were collected to culture. And the knockdown or overexpression efficiency were verified by WB assay.

Mouse xenograft model

All of mice were fed and housed in the Animal Institute of Laboratory Animal Centre Peking University according to the protocols approved by the Medical Experimental Animal Care Commission. All mice received humane care per the criteria outlined in the “Guide for the Care and Use of Laboratory Animals” issued by the National Institutes of Health (NIH publication 86-23 revised 1985).

For determination of the promoting tumor growth effect of PEX5 in vivo, nude mice (4-6 weeks of age, male, BALB/c) were applied. The stable overexpression plasmid (OE-PEX5 and the OE-PEX5 ctrl plasmid) and stable downregulation plasmid (sh-PEX5 and sh-PEX5 ctrl plasmid) were conducted. 4 x 106 stably transfected HepG2 cells during the exponential-growth phase of different experimental groups (5 mice per group) were collected and injected subcutaneously into the skin of back. After 4 weeks, mice were sacrificed and the subcutaneous tumor tissues were collected. Sketch maps were showed in Supplementary Fig.8A.

For determination of the inhibiting tumor growth effect of miR-31-5p *in vivo*, nude

mice (4-6 weeks of age, male, BALB/c), miR-31-5p agomir and agomir ctrl (Ribobio Biotech) were applied. 4 x 10^6 stably transfected HepG2 cells during the exponential-growth phase of different experimental groups (5 mice per group), were collected and injected subcutaneously into the skin of back. After 1 week, miR-31-5p agomir or miR-31-5p agomir ctrl was injected into the implanted tumor tissues at the dose of 1 nmol (in 20 μl phosphate-buffered saline) per mouse every 4 days. After 3 weeks, mice were sacrificed and the subcutaneous tumor tissues were removed. Sketch maps were showed in Supplementary Fig.8B.

And, all of the cancer size were calculated according to the formula [4] : cancer volume (mm^3) = ($L \times W^2$)/2, where L = long axis and W = short axis. A portion of the cancer tissues were fixed with paraformaldehyde, sectioned and performed with hematoxylin and eosin staining (HE) and the IHC for PEX5, Ki-67, MMP2 and MMP9. The rest of tumor tissues were stored in liquid nitrogen for WB.

For determination of the promoting tumor metastasis effect of PEX5 *in vivo*, nude mice (4-6 weeks of age, male, BALB/c) were applied. 4 x 10^6 /150 μl stably transfected HepG2 cells of different experimental groups (5 mice per group), including OE-PEX5, OE-PEX5 ctrl, sh-PEX5, and sh-PEX5 ctrl cells, were collected and injected via tail vein. For determination of the inhibiting tumor metastasis effect of miR-31-5p *in vivo*, miR-31-5p agomir and miR-31-5p agomir ctrl were applied. After incubation with miR-31-5p agomir or miR-31-5p agomir ctrl for three days in the dishes, 4 x 10^6 /150 μl stably transfected HepG2 cells of different experimental groups (5 mice per group), including OE-PEX5+ miR-31-5p agomir ctrl group, OE-PEX5+ miR-31-5p agomir group, sh-PEX5 ctrl + miR-31-5p agomir ctrl group and sh-PEX5 ctrl + miR-31-5p agomir group were collected and injected via tail vein. After 6 weeks, D-Luciferin (10 μl/g) was injected into abdominal cavity for 10-15 min. Then, the mice were imaged on

a Xenogen IVIS Lumina XRMS Series III live animal biophotonic imaging system (Caliper Life Sciences). Last, the lungs were removed, fixed, sectioned and performed with hematoxylin and eosin staining (HE). Sketch maps were showed in Supplementary Fig.8C-D.

For determination of the effect of PEX5 on HCC radiation sensitivity *in vivo*, 4 x 10^6 stably transfected HepG2 cells of different experimental groups, including sh-PEX5, and sh-PEX5 ctrl, were collected and injected subcutaneously into the skin of back of nude mice (4-6 weeks of age, male, BALB/c). When the average cancer volume achieved 5 mm x 5 mm x 5 mm [5], mice were randomly assigned to 4 groups as follows (5 mice per group):

1) sh-PEX5 ctrl group (0Gy),

2) sh-PEX5 group (0Gy),

3) sh-PEX5 ctrl+IR group (2Gy/day x 5 days with Varian Clinac 21EX(Trilogy)),

4) sh-PEX5+IR group (2Gy/day x 5 days with Varian Clinac 21EX(Trilogy)).

Then, after 4 weeks, mice were sacrificed and the subcutaneous tumor tissues were removed, fixed and stored at -80°C to make further experiments. Sketch maps were showed in Supplementary Fig.8E-F.

For determination of the effect of miR-31-5p on HCC radiation sensitivity *in vivo*, miR-31-5p agomir and miR-31-5p agomir ctrl were applied. 4 x 10^6 stably transfected sh-PEX5 ctrl cells and HepG2 R-C, were collected and injected subcutaneously into the skin of back of nude mice (4-6 weeks of age, male, BALB/c). When the average cancer volume achieved 5 mm x 5 mm x 5 mm, mice were randomly assigned to 5 groups as follows (5 mice per group):

1) sh-PEX5 ctrl +miR-31-5p agomir ctrl group,

2) sh-PEX5 ctrl +miR-31-5p agomir group,

3) sh-PEX5 ctrl +miR-31-5p agomir +IR group (2Gy/day x 5 days, miR-31-5p agomir 1 nmol/4days),
4) R-C +miR-31-5p agomir ctrl +IR group (2Gy/day x 5 days, miR-31-5p agomir ctrl 1 nmol/4days),
5) R-C +miR-31-5p agomir +IR group (2Gy/day x 5 days, miR-31-5p agomir 1 nmol/4days).

miR-31-5p agomir and miR-31-5p agomir ctrl (Ruibo Biotech,Guangzhou, China) were injected into tumor tissues at the dose of 1 nmol (in 20 μl phosphate-buffered saline) per mouse every 4 days. And all of the tumor tissues of mice were exposed to radiation of 2Gy/day x 5 days. Then, after 4 weeks, mice were sacrificed and the subcutaneous tumor tissues were removed, fixed and stored at -80°C to make further experiments. Sketch maps were showed in Supplementary Fig.8G-I.

Patients and tissue microarray

Paired tissue samples including cancer tissues and para-cancerous tissues of 12 HCC patients who had undergone surgical resection were provided friendly by Beijing Cancer Hospital. Informed consents were obtained and approved by the ethics committee of Peking University School of Oncology (Beijing Cancer Hospital and Institute).

To validate the clinical significance of miR-31-5p and PEX5 in HCC, we purchased two tissue microarrays from Outdo Biotech Co, Ltd. (Shanghai, China). One of them contains 90 pairs of HCC cancer tissues and adjacent normal tissues, another has 87 pairs. Immunohistochemistry (IHC) was used to detect the expression of PEX5, and in situ hybridization (ISH) was performed to validate the expression of miR-31-5p. The quantitation of immunostaining score for miR-31-5p and PEX5 were completed by three independent researchers who were blinded relative detail information. The

immunostaining score was calculated on the basis of the positive cells percentage and staining density with 12 standard points. The percentage of positive cells score was divided into five grades (percentage scores): 0 point, <5%; 1 point, 10-25%; 2 points, 26-50%; 3 points, 51-75%; and 4 points, >75%. The staining intensity was scored according to the staining characteristics of the target cells: 0 point (none), 1 point (low), 2 points (medium), 3 points (high). The total scores (percentage score x intensity score) ranged from 0 to 12. PEX5 expression was classified into low expression (≤8) or high expression (8-12) groups. The expression of miR-31-5p was divided into low expression (≤4) or high expression (4-12) groups. Nucleus PEX5 expression was classified into low expression (≤20%) or high expression (>20%) groups.

We confirmed miR-31-5p expression between HCC tissues and adjacent tissues with ISH on HCC tissues microarrays. Because 2 cases were broken, 175 HCC cases were enrolled in our study with the information in Supplementary Table 1. We validated PEX5 expression between HCC tissues and para-cancerous tissues with IHC on HCC tissues microarrays. For 8 cases has been broken, 169 HCC cases were enrolled in our study with the information in Supplementary Table 2.

Cycloheximide (CHX) chase assay

HepG2 and HLE cell lines were transfected for 48 h, then, the cells were incubated with DMEM containing 10% FBS and 100ug/mL [6] CHX. Next, at different time points, cells were harvested for WB assay.

ROS measurement

A ROS detection kit (Beyotime Biotechnology) was used to measure the ROS level in cells. DCFH-DA was diluted with serum-free medium at 1:1000 to a final concentration of 10 mmol/L. At 4 h after radiation, the cells were collected and suspended in the diluted DCFH-DA at a cell concentration of 1-20 x 10^6 / mL, and incubated in a 37 °C

cell incubator for 20 min.Then, we mixed upside down every 3-5 min to fully mix the probe and the cells. The cells were washed three times with serum-free cell culture medium to sufficiently remove DCFH-DA which did not enter the cells. And flow cytometry (BD FACS Calibur, 488nm of excitation wavelength and 525nm of emission wavelength) was applied to detect the ROS level.

Apoptosis assay

Allophycocyanin (APC)-conjugated Annexin V and Propidium Iodide (PI) kit (KGA1030-50) was used to detect the apoptotic cells according to the manufacturer's instructions. HepG2 and HLE cells were transfected with miR-31-5p mimics and si-PEX5 for 48 h, then exposed to irradiation of 10 Gy. After 48 h, the cells were harvested with trypsin and washed twice with PBS. Cells were then resuspended in 1x binding buffer at a concentration of 1 x 10^6 cells/ml. Next, 100 μl of the solution (1 x 10^5 cells) was transfered to a 5 ml culture tube and 5 μl of APC Annexin V and 5 μl PI were added into the solution. The mixture was incubated for 15 min at RT (25°C) in the dark. 200 μl of 1X Binding Buffer was added to each tube and the apoptosis rate of the cells was analyze by flow cytometry within 1 h.

Online databases

ONCOMINE database: https://www.oncomine.org/[7]

Ualcan database: http://ualcan.path.uab.edu/[8]

GEO database: https://www.ncbi.nlm.nih.gov/gds/[9]

Linkedomics database: http://www.linkedomics.org/login.php[10]

The cancer genome atlas: http://www.tcga.org/

GEPIA database: http://gepia.cancer-pku.cn/index.html[11]

Kmplot database: http://kmplot.com/analysis/index.php[12]

MiRWalk database: http://zmf.umm.uni-heidelberg.de/apps/zmf/mirwalk2/[13]

HCCDB: http://lifeome.net/database/hccdb/home.html[14]

Supplementary Table 1. Clinical characteristics of patients enrolled in the present study (enrolled 175 patients).

Clinicopathological variable	Value
Sex (%)	
Male	89.14%
Female	10.86%
Median age, years (range)	53(25-78)
Tumor size (%)	
≤2	9.71%
2-5	43.43%
≥5	46.86%
Vascular invasion (%)	
Positive	17.14%
Negative	82.86%
Lymph node metastasis (%)	
Positive	1.14%
Negative	98.86%
Histology grade (%)	
I	7.43%
II	72%
III	20.57%
Cirrhosis	
Positive	63.43%
Negative	36.57%
AJCC 7th T stage (%)	
T1	41.71%
T2	23.44%
T3	33.14%
T4	1.71%

Supplementary Table 2. Clinical characteristics of patients enrolled in the present study (enrolled 169 patients).

Clinicopathological variable	Value
Sex (%)	
Male	89.35%
Female	10.65%
Median age, years (range)	55(27-78)
Tumor size (%)	
≤2	10.06%
2-5	42.60%
≥5	47.34%
Vascular invasion (%)	
Positive	17.76%
Negative	82.24%
Lymph node metastasis (%)	
Positive	1.18%
Negative	98.82%
Histology grade (%)	
I	7.1%
II	72.19%
III	20.71%
Cirrhosis	
Positive	63.31%
Negative	36.69%
AJCC 7th T stage (%)	
T1	42.01%
T2	23.07%
T3	33.14%
T4	1.78%

Supplementary Table 3. Associations between PEX5 and miR-31-5p expression and clinicopathological features.

	PEX5 expression			miR-31-5p expression		
Clinicopathological variable	High	Low	χ2/P-value	High	Low	χ2/P-value
Sex			6.904			0.482
Male	112	39	/	61	95	/
Female	8	10	0.008	9	10	0.487
Age at diagnosis, years			0.265			0.963
≤45	108	31	/	59	78	/
>45	22	8	0.607	13	25	0.326
Tumor size, cm						
≤2	9	8	2.358	7	10	5.281
2-5	51	21	/	28	48	/
>5	57	23	0.308	46	36	0.047
Vascular invasion			3.999			4.5291
Positive	17	13	/	11	19	/
Negative	104	35	0.046	84	61	0.033
Lymphnode metastasis			0.850			1.381
Positive	2	0	/	0	2	/
Negative	117	50	0.356	71	102	0.240
Histology grade						
I	5	7	6.304	4	9	1.855
II	86	36	/	50	76	/
III	28	7	0.043	18	18	0.396
Cirrhosis			0.118			0.049
Positive	75	32	/	47	64	/
Negative	45	17	0.731	26	38	0.824
AJCC 7th T stage (%)						
T1	48	23	2.922	38	35	8.1747
T2	29	10	/	11	30	/
T3	35	21	0.403	20	38	0.042
T4	3	0		2	1	

Note: PEX5 had significant correlation with vascular invasion (P=0.046) and histology grade (P=0.043) of HCC patients. miR-31-5p had significant correlation with the tumor size (P=0.047), vascular invasion (P=0.033) and TNM stage (P=0.042) of HCC patients.

Supplementary Table 4. Incidence of lung metastasis in transplanted nude mice.

(χ2 Test)

Incidence of lung metastasis in transplanted nude mice		
Groups	Lung metastasis rate	P value
OE-PEX5	5/5	0.009823275
OE-PEX5 ctrl	1/5	
OE-PEX5+miR-31-5p agomir ctrl	5/5	0.03843393
OE-PEX5+miR-31-5p agomir	2/5	

Supplementary Table 5. Sequence of primers used in qRT-PCR

Target	Primer sequence
miR-31-5p	Forward: 5'-GCGGAGGCAAGATGCTGGCA-3'<br>Reverse: universal primer
U6	Forward: 5'-CTCGCTTCGGCAGCACA-3'<br>Reverse: 5'-AACGCTTCACGAATTTGCGT-3'
GAPDH	Forward: 5'-GGAGCGAGATCCCTCCAAAAT-3'<br>Reverse: 5'-GGCTGTTGTCATACTTCTCATGG-3'
PEX5	Forward: 5'- AGCAGATTGAGCAGTCAAACTT-3'<br>Reverse: 5'- TTGGGACCAGTCAGTCTCATT-3'
β-Catenin	Forward: 5'- TGGATTGATTCGAAATCTTGCC-3'<br>Reverse: 5'- GAACAAGCAACTGAACTAGTCG-3'

Supplementary Table 6: antibodies for WB, IHC and IF

Antibody NAME	Brand	Application and Condition	Cat number
PEX5	Cell Signaling Technology	WB (1:1000)	#83020
PEX5	Proteintech	Co-IP (4μg/mL)<br>IHC (1:200)	12545-1-AP
β-catenin	Cell Signaling Technology	WB (1:1000)<br>IF (1:100)<br>Co-IP (1:50)	#8480
ATM	GeneTex	WB (1:1000)<br>Co-IP (6μg/mL)	GTX70103
Anti-ATM (phosphor S1981)	Abcam	WB (1:1000)<br>IF (1:200)<br>Co-IP (4μg/mL)	ab19304
Phospho-Histone H2A.X (Ser139/Tyr142)	Cell Signaling Technology	WB (1:1000)<br>IF (1:100)<br>Co-IP (1:50)	#5438
Phospho-Histone H2A.X (Ser139)	Cell Signaling Technology	IF (1:100)	#80312
Ku70	Proteintech	WB (1:1000)	10723-1-AP
Ku80	Proteintech	WB (1:1000)	16389-1-AP
MMP2	Proteintech	WB (1:300)<br>IHC (1:200)	10373-2-AP
MMP9	Proteintech	WB (1:400)<br>IHC (1:200)	10375-2-AP
Histone H2A.X	Proteintech	WB (1:1000)	10856-1-AP
E-cadherin	Proteintech	WB (1:1000)	20874-1-AP
N-cadherin	Cell Signaling Technology	WB (1:400)	#13116
BRCA1	Abcam	WB (1:1000)	ab131360
Chk1	Proteintech	WB (1:1000)	25887-1-AP
P-Chk1(Ser317)	Cell Signaling Technology	WB (1:1000)	12302s
GAPDH	Proteintech	WB (1:5000)	60004-1-Ig
JNK2	Proteintech	WB (1:1000)<br>Co-IP 4μg/mL)	51153-1-AP
Rac1	Proteintech	WB (1:1000)	24072-1-AP

		Co-IP (μg/mL)	
Vimentin	Proteintech	WB (1:1000)	10366-1-AP
SNAI1	Proteintech	WB (1:1000)	13099-1-AP
Caspase-3	Cell Signaling Technology	WB (1:1000)<br>IHC (1:1000)	#9662
Bax	Cell Signaling Technology	WB (1:1000)	#5023
Bcl-2	Cell Signaling Technology	WB (1:1000)	#3498
Lamin B1	Proteintech	WB 1:1000)	12987-1-AP
a-tubulin	SANTA CRUZ	WB 1:200)	sc-5286
Phospho-β-Catenin (Ser33/37/Thr41)	Cell Signaling Technology	WB (1:500)	#9561
KI67	Proteintech	IHC (1:5000)	27309-1-AP
Anti-rabbit IgG, (H+L), F(ab')2 Fragment (Alexa Fluor® 594 Conjugate)	Cell Signaling Technology	IF (1:1000)	#8889
Alexa Fluor® 488 – Conjugated Goat anti-Mouse IgG （H+L）	ZSGB-BIO	IF (1:200)	ZF-0512
HRP-labeled Goat Anti-Rabbit IgG (H+L)	Beyotime Biotechnology	WB (1:1000)	A0208
HRP-labeled Goat Anti-Mouse IgG (H+L)	Beyotime Biotechnology	WB (1:1000)	A0216
DAPI	Solarbio Science	IF (1:100)	C0060

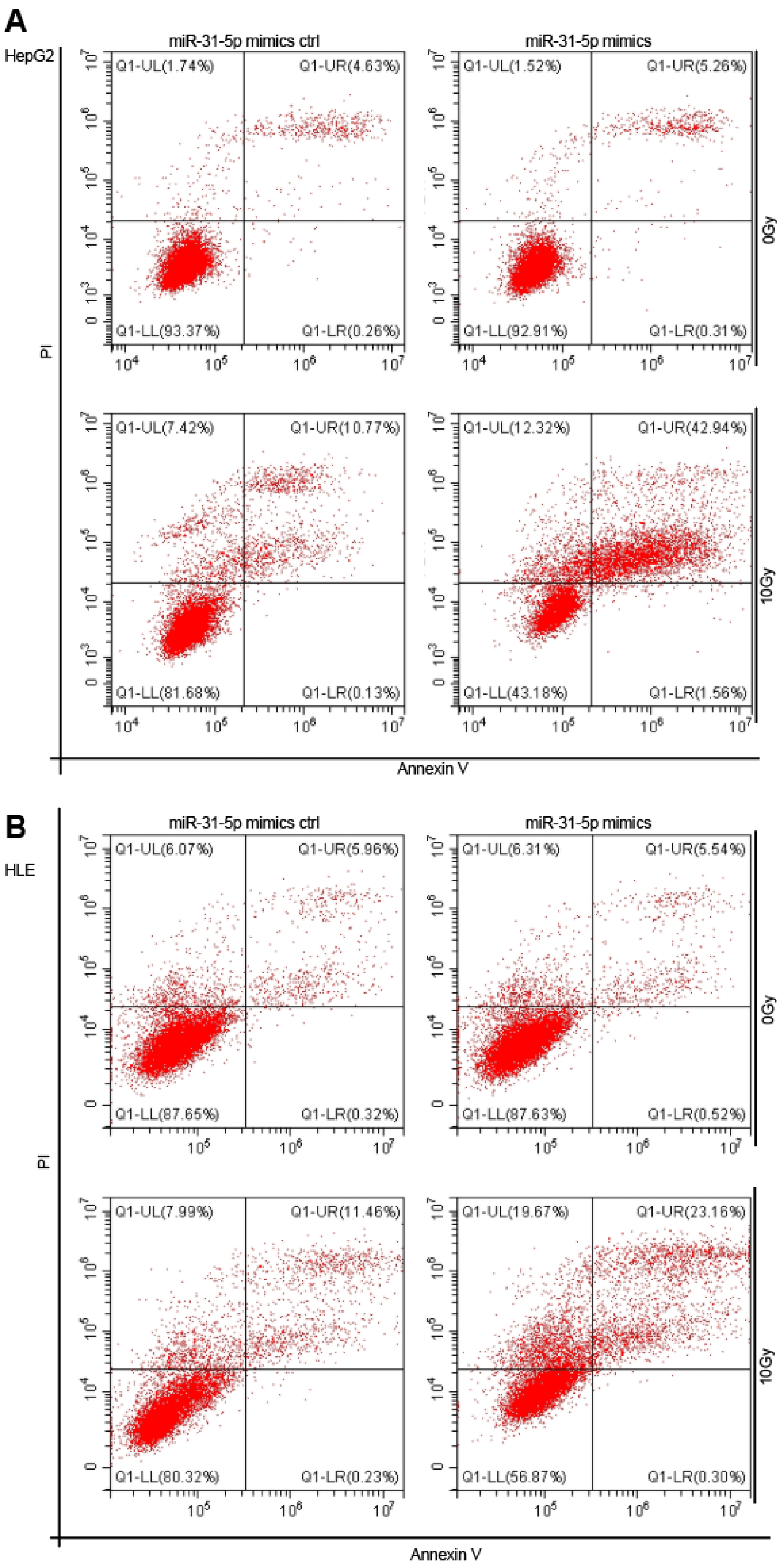

Supplementary Figure.1. miR-31-5p increase the apoptosis rate of HepG2 and HLE cell lines upon radiation. (A) miR-31-5p increase the apoptosis rate of HepG2

cells upon radiation. (B) miR-31-5p increase the apoptosis rate of HLE cells upon radiation. Results are representative of three independent experiments. Abbreviations: ctrl, control; PI, Propidium Iodide.

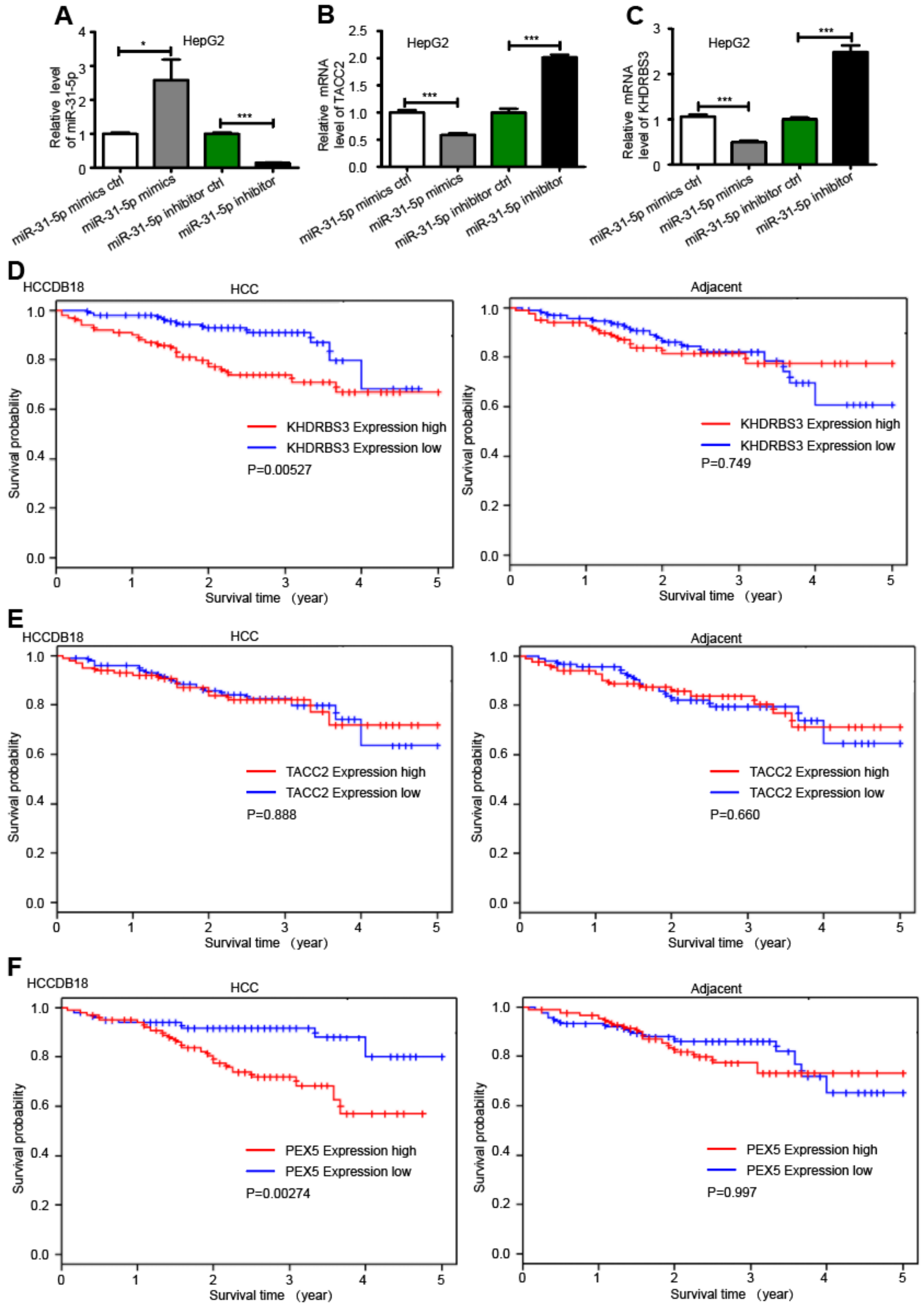

Supplementary Figure.2. The potential targets of miR-31-5p in HCC. (A-C) The mRNA level of TACC2 and KHDRBS3 could be reduced by miR-31-5p mimics (*P <0.05, ***P<0.001). Results are representative of three independent experiments. (D)

The overall survival of HCC patients with different level of KHDRBS3 (HCCDB database, Kaplan-Meier survival analysis). (E) The overall survival of HCC patients with different level of TACC2 (HCCDB database, Kaplan-Meier survival analysis). (F) The overall survival of HCC patients with different level of PEX5 (HCCDB database, Kaplan-Meier survival analysis). Abbreviations: ctrl, control.

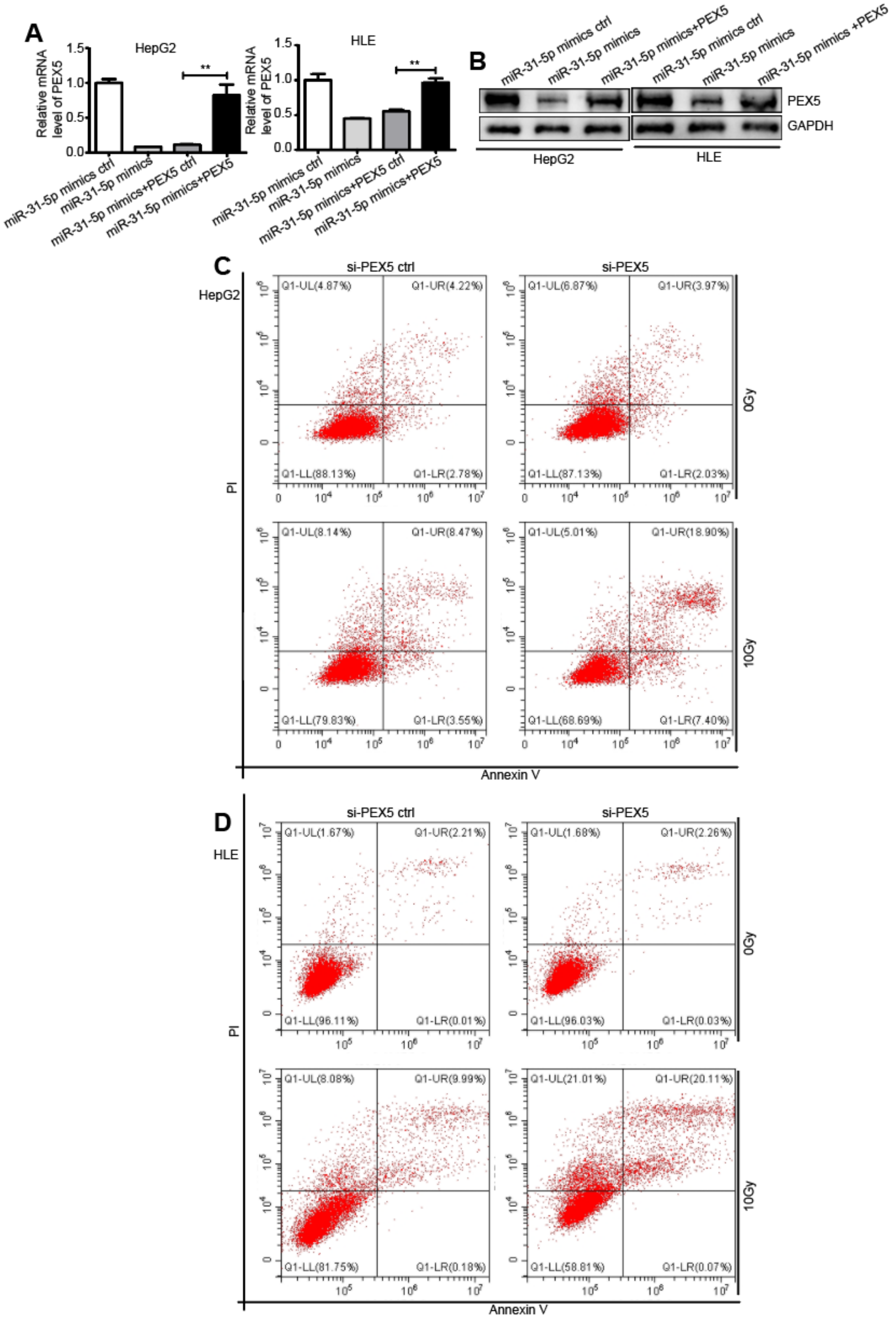

Supplementary Figure.3. si-PEX5 increase the apoptosis rate of HepG2 and HLE cell lines upon radiation. (A) miR-31-5p decreased PEX5 level could be reversed by

PEX5 plasmid transfection after 48h at mRNA level (**P<0.01). (B) miR-31-5p decreased PEX5 level could be reversed by PEX5 plasmid transfection after 48h at protein level. (C) si-PEX5 increase the apoptosis rate of HepG2 cells upon radiation. (D) si-PEX5 increase the apoptosis rate of HLE cells upon radiation. Results are representative of three independent experiments. Abbreviations: GAPDH, glyceraldehyde 3-phosphate dehydrogenase; ctrl, control; mut, mutant; NC, negative control; PI, Propidium Iodide.

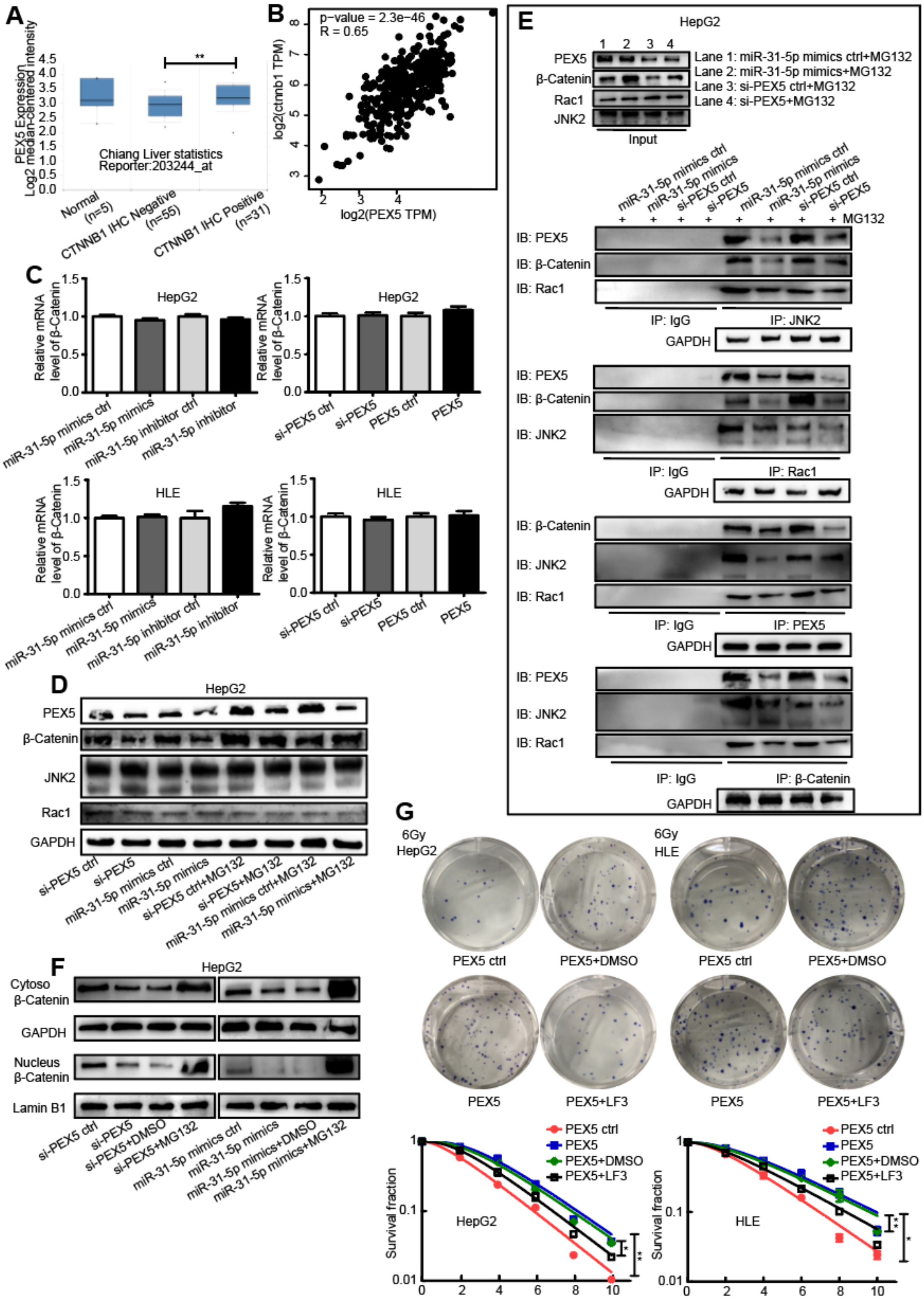

Supplementary Figure.4. PEX5 activated WNT/β-catenin pathway in HCC. (A) The ONCOMINE data about the relationship between PEX5 and CTNNB1 (the gene encoding β-catenin).The patients with CTNNB1 IHC positive staining have higher

PEX5 protein expression than those with CTNNB1 IHC negative staining. (**$P<0.01$). (B) The correlation between PEX5 and CTNNB1 in HCC in the GEPIA database. PEX5 had positive correlationship with CTNNB1 in HCC. ($p=2.3e-46$, Spearman Correlation analysis). (C) The mRNA level of β-catenin in HepG2 and HLE cell lines with different miR-31-5p and PEX5 level. (D) The protein level of PEX5、β-catenin、JNK2 and Rac1 in HepG2 cells with different miR-31-5p and PEX5 level with or without MG132 treatment (20 μM for 4h). MG132 reversed the si-PEX5 and miR-31-5p mimics suppressed β-catenin level. (E) The interaction among Rac1, JNK2 and β-catenin in HepG2 cells with different miR-31-5p and PEX5 level after MG132 treatment (20 μM for 4h). The interaction among Rac1, JNK2 and β-catenin in HepG2 cell was inhibited by si-PEX5 and miR-31-5p mimics though with MG132 treatment (20 μM for 4h). (F) The nuclear β-catenin and cytosol β-catenin protein level in HepG2 cells with different miR-31-5p and PEX5 level after MG132 treatment (20 μM for 4h). The miR-31-5p mimics and si-PEX5 decreased nuclear β-catenin and cytosol β-catenin protein level could be rescued by MG132. (G) PEX5 overexpression promotes the radioresistance of HepG2 and HLE cell lines and LF3 (Selleck) can rescue the effect of PEX5 overexpression. For LF3 treatment, cells were treated with LF3 (30 μM) for 24 h and then exposed to radiation of 0 Gy, 2 Gy, 4 Gy, 6 Gy, 8 Gy and 10 Gy. Top: the representative images of clone formation of cells exposed to the radiation of 6 Gy. Bottom: the statistical analysis results (*$P<0.05$, **$P<0.01$). Results are representative of three independent experiments. Abbreviations: GAPDH, glyceraldehyde 3-phosphate dehydrogenase; ctrl, control; si-PEX5, siRNA-PEX5; DMSO, Dimethyl

Sulfoxide.

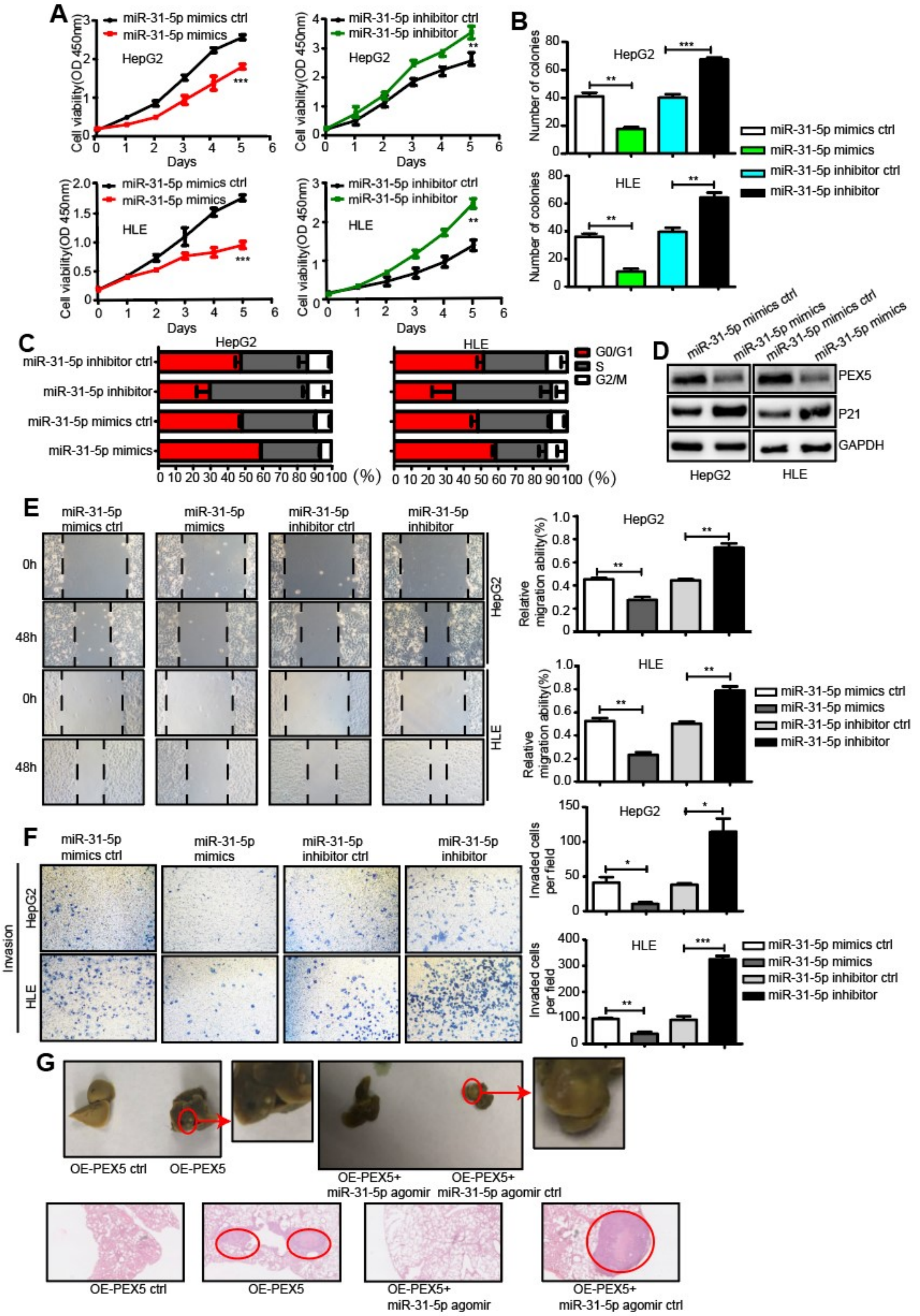

Supplementary Figure.5. miR-31-5p inhibited the growth, migration and invasion in HepG2 and HLE cell lines *in vitro* and *in vivo*. (A) The effects of miR-31-5p on

HCC cell lines growth validated by CCK8 (**P < 0.01, ***P < 0.001). The growth of HepG2 and HLE cell lines were inhibited by miR-31-5p mimics and enhanced by miR-31-5p inhibitor. (B) The effects of miR-31-5p on HCC cell lines colony formation ability (**P < 0.01, ***P < 0.001). The colony formation ability of HepG2 and HLE cell lines were inhibited by miR-31-5p mimics and enhanced by miR-31-5p inhibitor. (C) miR-31-5p mimics increased the ratio of cell in the G1 phase with a concomitant reduction of cell ratio in the S phase and G2/M phase in both the cell lines, however, miR-31-5p inhibitor reversed this trend. Results are representative of three independent experiments. (D) miR-31-5p mimics increased the protein level of $p21^{WAF1/Cip1}$ in both the cell types. Results are representative of three independent experiments. (E) The effect of miR-31-5p on HCC cell lines migration ability (**P < 0.01). The migration abilities of HepG2 and HLE cell lines were inhibited by miR-31-5p mimics and enhanced by miR-31-5p inhibitor. Results are representative of three independent experiments. (F) The effect of miR-31-5p on HCC cell lines on the cell invasion ability (*P < 0.05, **P < 0.01, ***P < 0.001). The invasion abilities of HepG2 and HLE cell lines were inhibited by miR-31-5p mimics and enhanced by miR-31-5p inhibitor. Results are representative of three independent experiments. (G) Incidence of lung metastasis among the different groups of nude mice. Hematoxylin and eosin–stained images of lung tissues (n=5). Abbreviations: GAPDH, glyceraldehyde 3-phosphate dehydrogenase; ctrl, control; sh-PEX5, shRNA-PEX5; OE-PEX5, Overexpressed-PEX5.

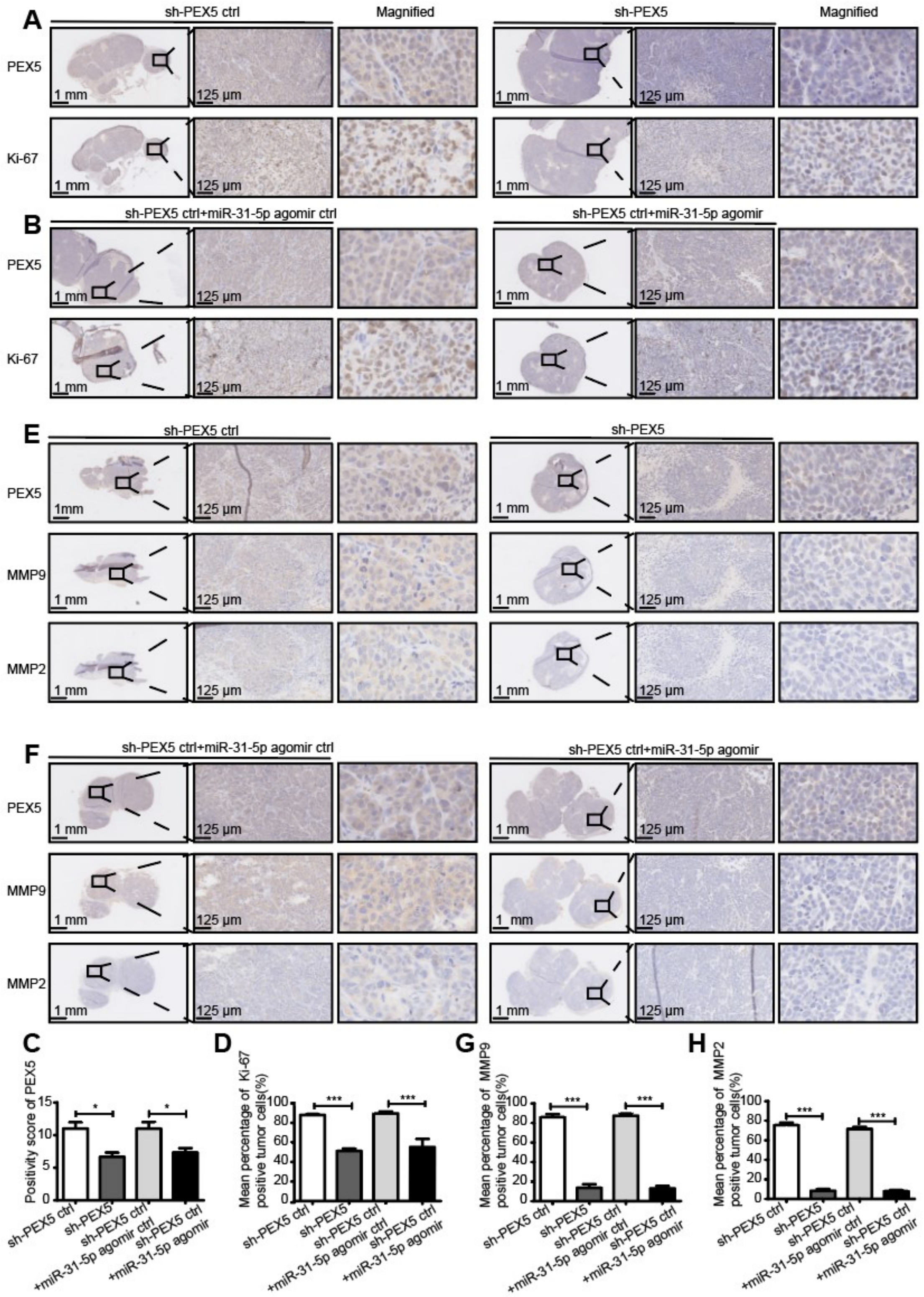

Supplementary Figure.6. PEX5 increased the Ki-67, MMP2 and MMP9 expression level in vivo, and the effects were inhibited by miR-31-5p. (A-D) Compared to the control group, lower level of Ki-67 can be observed in the mice of

PEX5 downregulating group, and this tendency was reversed by miR-31-5p agomir. Note: Each image represented the mean of one xenografts tumor section calculating in 10 high-power fields (*P<0.05, ***P<0.001). (E-H) Compared to the control group, lower mean percentage of positive cell of MMP2 and MMP9 could be observed in the mice of PEX5 downregulating group, and this tendency could be reversed by miR-31-5p agomir (***P<0.001). Abbreviations: sh-PEX5, shRNA-PEX5; ctrl, control.

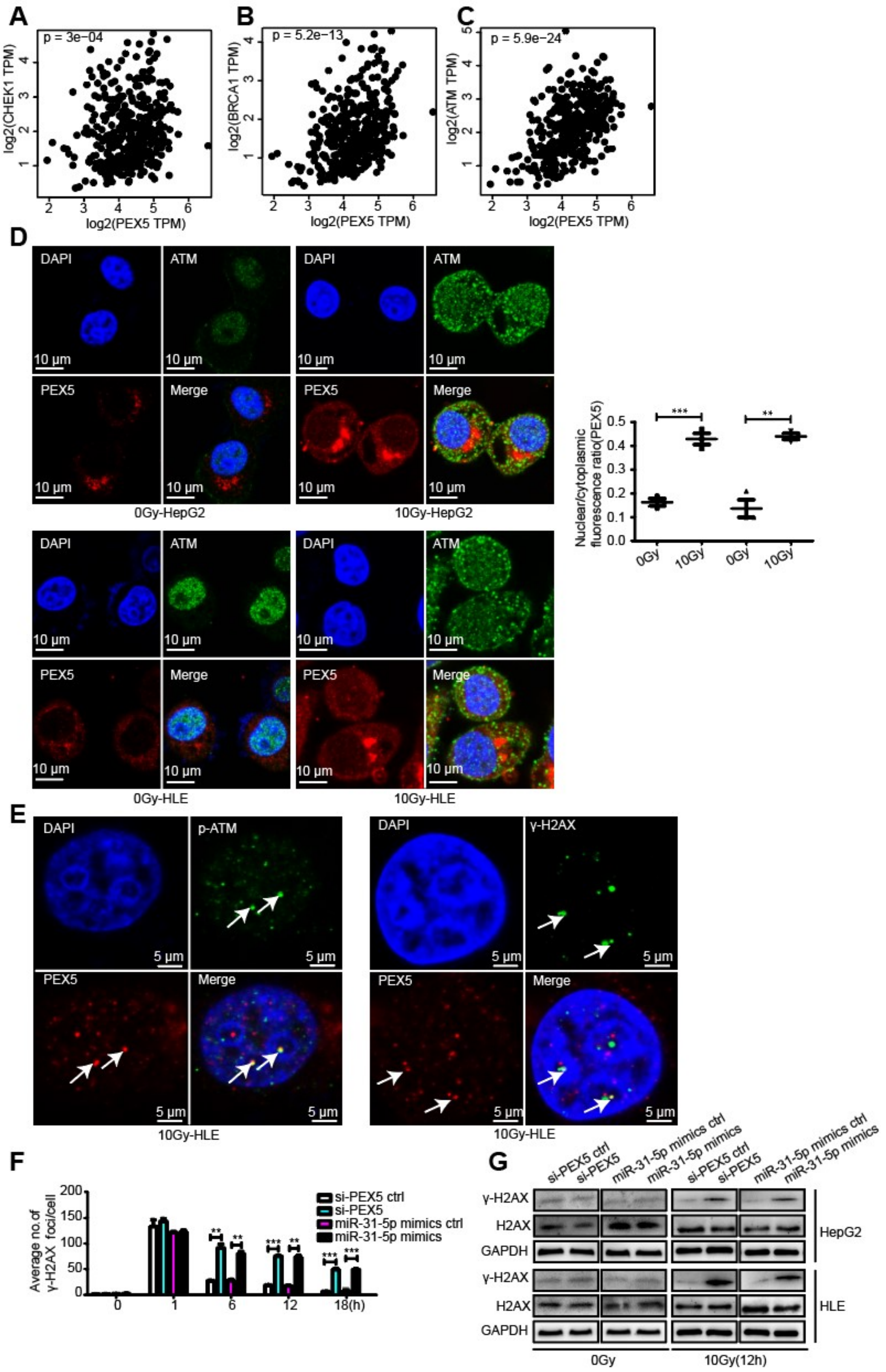

Supplementary Figure.7. PEX5 may be involved in the DNA damage repair process. *PEX5* had positive correlation with *CHEK1* (A), *BRCA1* (B) and *ATM* (C) in

HCC. The data was from GEPIA database. (Spearman Correlation analysis). (D) The representative images of the nucleus PEX5 intensity with or without radiation in HepG2 and HLE cells were showed at left, with the data analysis result at right. Upon radiation, the nucleus intensity of PEX5 was elevated. PEX5 was labelled with Alexa Fluor®594 in Red, the ATM with FITC 488 in green and the nucleus with DAPI in blue. (n=30, **P<0.01, ***P<0.001). (E) Upon radiation, PEX5 may colocalized with P-ATM and γ-H2AX foci. PEX5 was labelled with Alexa Fluor®594 in Red, the P-ATM or γ-H2AX with FITC 488 in green and the nucleus with DAPI in blue. Results are representative of three independent experiments. (F) Quantification of the number of γ-H2AX foci in each nucleus at different time points after radiation. A number of γ-H2AX foci with no significant difference in quantity can be observed at 1 h in nucleus of cells among groups. However, after radiation, the average number of γ-H2AX foci was markedly higher in cells transfected with miR-31-5p mimics or si-PEX5 than that in the cells of control groups at 6 h, 12 h and 18 h. Over 50 cells were randomly picked for quantification. (n≥50, **P<0.01, ***P<0.001). (G) Upon radiation, knockdown of PEX5 or upregulation of miR-31-5p could increased the protein level of γ-H2AX in the both cell types. Results are representative of three independent experiments. Abbreviations: si-PEX5, siRNA-PEX5; ctrl, control; GAPDH, glyceraldehyde 3-phosphate dehydrogenase.

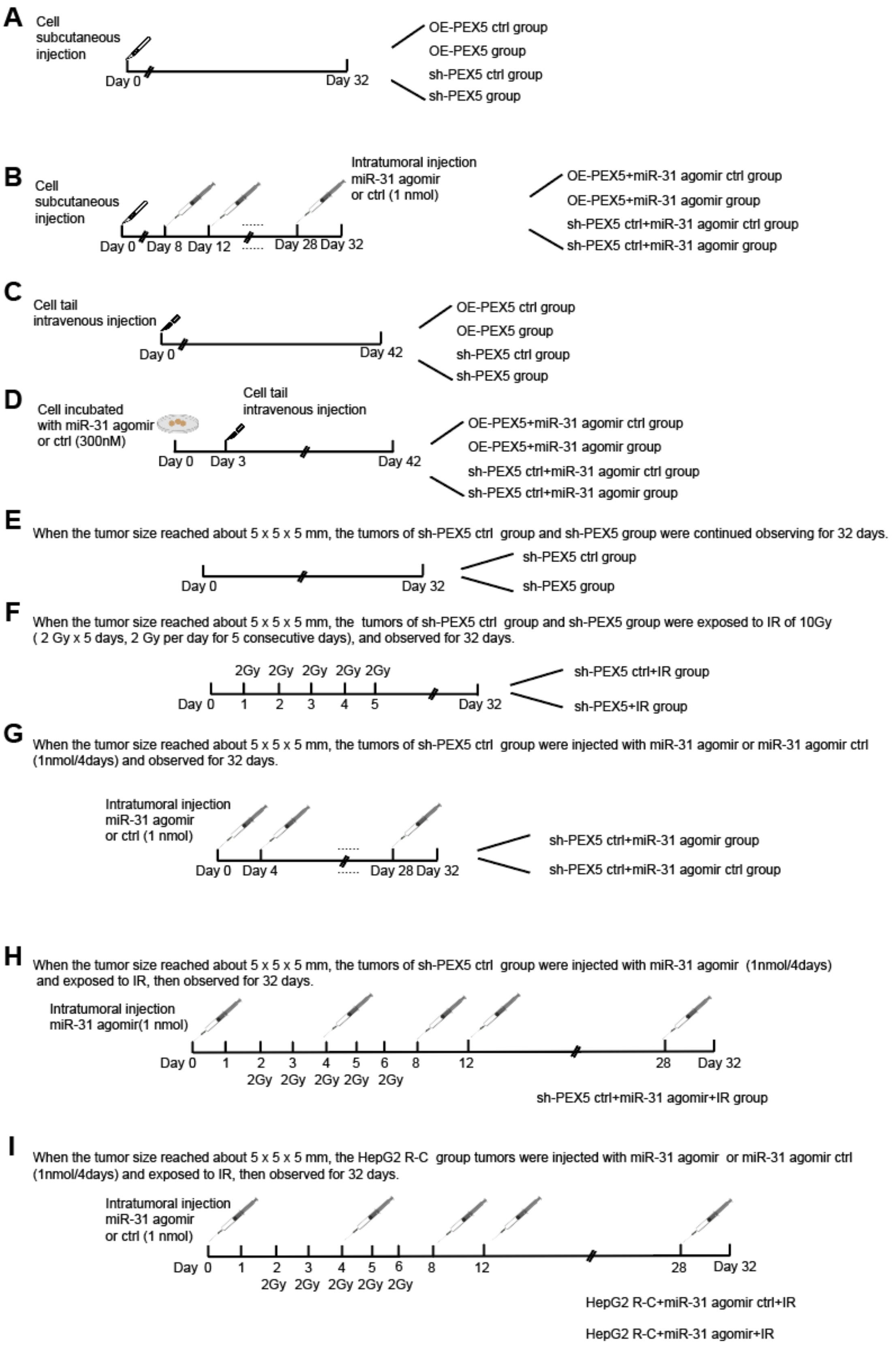

Supplementary Figure.8. Mice model establishment strategies in various groups.

(A) To detect the effect of PEX5 on the tumor growth *in vivo* (n=5). (B) To detect the

effect of miR-31-5p on the tumor growth *in vivo* (n=5). (C) To detect the effect of PEX5 on the tumor metastasis *in vivo* (n=5). (D) To detect the effect of miR-31-5p on the tumor metastasis *in vivo* (n=5). (E-F) To detect the effect of PEX5 on the radiation sensitivity *in vivo* (n=5). (G-I) To detect the effect of miR-31-5p on the radiation sensitivity *in vivo* (n=5). Abbreviations: sh-PEX5, shRNA-PEX5; ctrl, control; R-C, radioresistant cell; OE-PEX5, Overexpress-PEX5; sh-PEX5, shRNA-PEX5; IR, irradiation.

Supplementary references

1. Chaoui D, Faussat AM, Majdak P, Tang R, Perrot JY, Pasco S, et al. JC-1, a sensitive probe for a simultaneous detection of P-glycoprotein activity and apoptosis in leukemic cells. Cytometry Part B, Clinical cytometry. 2006; 70: 189-96.
2. Zeng J, Zhao H, Chen B. DJ-1/PARK7 inhibits high glucose-induced oxidative stress to prevent retinal pericyte apoptosis via the PI3K/AKT/mTOR signaling pathway. Exp Eye Res. 2019; 189: 107830.
3. Savitskiy VP, Shman TV, Potapnev MP. Comparative measurement of spontaneous apoptosis in pediatric acute leukemia by different techniques. Cytometry Part B, Clinical cytometry. 2003; 56: 16-22.
4. Naito S, von Eschenbach AC, Giavazzi R, Fidler IJ. Growth and metastasis of tumor cells isolated from a human renal cell carcinoma implanted into different organs of nude mice. Cancer Res. 1986; 46: 4109-15.
5. Luo J, Zhu W, Tang Y, Cao H, Zhou Y, Ji R, et al. Artemisinin derivative artesunate induces radiosensitivity in cervical cancer cells in vitro and in vivo. Radiat Oncol. 2014; 9: 84.
6. Liu S, Wang J, Shao T, Song P, Kong Q, Hua H, et al. The natural agent rhein induces beta-catenin degradation and tumour growth arrest. J Cell Mol Med. 2018; 22: 589-99.
7. Rhodes DR, Yu J, Shanker K, Deshpande N, Varambally R, Ghosh D, et al. ONCOMINE: a cancer microarray database and integrated data-mining platform. Neoplasia. 2004; 6: 1-6.
8. Chandrashekar DS, Bashel B, Balasubramanya SAH, Creighton CJ, Ponce-Rodriguez I, Chakravarthi B, et al. UALCAN: A Portal for Facilitating Tumor Subgroup Gene Expression and Survival Analyses. Neoplasia. 2017; 19: 649-58.
9. Barrett T, Wilhite SE, Ledoux P, Evangelista C, Kim IF, Tomashevsky M, et al. NCBI GEO: archive for functional genomics data sets--update. Nucleic Acids Res. 2013; 41: D991-5.
10. Vasaikar SV, Straub P, Wang J, Zhang B. LinkedOmics: analyzing multi-omics data within and across 32 cancer types. Nucleic Acids Res. 2018; 46: D956-d63.
11. Tang Z, Li C, Kang B, Gao G, Li C, Zhang Z. GEPIA: a web server for cancer and normal gene expression profiling and interactive analyses. Nucleic Acids Res. 2017; 45: W98-w102.
12. Gyorffy B, Lanczky A, Szallasi Z. Implementing an online tool for genome-wide validation of survival-associated biomarkers in ovarian-cancer using microarray data from 1287 patients. Endocr Relat Cancer. 2012; 19: 197-208.
13. Dweep H, Sticht C, Pandey P, Gretz N. miRWalk--database: prediction of possible miRNA binding sites by "walking" the genes of three genomes. J Biomed Inform. 2011; 44: 839-47.
14. Lian Q, Wang S, Zhang G, Wang D, Luo G, Tang J, et al. HCCDB: A Database of Hepatocellular Carcinoma Expression Atlas. Genomics Proteomics Bioinformatics. 2018; 16: 269-75.
